# miRNA Genetic Variants Alter Their Secondary Structure and Expression in Patients With RASopathies Syndromes

**DOI:** 10.3389/fgene.2019.01144

**Published:** 2019-11-13

**Authors:** Joseane Biso de Carvalho, Guilherme Loss de Morais, Thays Cristine dos Santos Vieira, Natana Chaves Rabelo, Juan Clinton Llerena, Sayonara Maria de Carvalho Gonzalez, Ana Tereza Ribeiro de Vasconcelos

**Affiliations:** ^1^Bioinformatics Laboratory (LABINFO), National Laboratory for Scientific Computing (LNCC), Petrópolis, Brazil; ^2^Laboratory of Genomic Medicine, National Institute of Women, Children and Adolescents Health Fernandes Figueira (IFF/FIOCRUZ), Rio de Janeiro, Brazil; ^3^Department of Medical Genetics, National Institute of Women, Children and Adolescents Health Fernades Figueira, Oswaldo Cruz Foundation (Fiocruz), Rio de Janeiro, Brazil

**Keywords:** whole-exome-sequencing, miRNA, variants, RAS-MAPK, RASopathies

## Abstract

RASopathies are a group of rare genetic diseases caused by germline mutations in genes involved in the RAS–mitogen-activated protein kinase (RAS-MAPK) pathway. Whole-exome sequencing (WES) is a powerful approach for identifying new variants in coding and noncoding DNA sequences, including miRNAs. miRNAs are fine-tuning negative regulators of gene expression. The presence of variants in miRNAs could lead to malfunctions of regulation, resulting in diseases. Here, we identified 41 variants in mature miRNAs through WES analysis in five patients with previous clinical diagnosis of RASopathies syndromes. The pathways, biological processes, and diseases that were over-represented among the target genes of the mature miRNAs harboring variants included the RAS, MAPK, RAP1, and PIK3-Akt signaling pathways, neuronal differentiation, neurogenesis and nervous system development, congenital cardiac defects (hypertrophic cardiomyopathy, dilated cardiomyopathy, and arrhythmogenic right ventricular cardiomyopathy), and the phenotypes and syndromes of RASopathies (Noonan syndrome, Legius syndrome, Costello syndrome, Cafe au lait spots multiple, subaortic stenosis, pulmonary valve stenosis, and LEOPARD syndrome). Furthermore, eight selected variants in nine mature miRNAs (hsa-miR-1304, hsa-miR-146a, hsa-miR-196a2, hsa-miR-499a/hsa-miR-499b, hsa-miR-449b, hsa-miR-548l, hsa-miR-575, and hsa-miR-593) may have caused alterations in the secondary structures of miRNA precursor. Selected miRNAs containing variants such as hsa-miR-146a-3p, hsa-miR-196a-3p, hsa-miR-548l, hsa-miR-449b-5p, hsa-miR-575, and hsa-miR499a-3p could regulate classical genes associated with Rasopathies and RAS-MAPK pathways, contributing to modify the expression pattern of miRNAs in patients. RT-qPCR expression analysis revealed four differentially expressed miRNAs that were downregulated: miRNA-146a-3p in P1, P2, P3, P4, and P5, miR-1304-3p in P2, P3, P4, and P5, miR-196a2-3p in P3, and miR-499b-5p in P1. miR-499a-3p was upregulated in P1, P3, and P5. These results indicate that miRNAs show different expression patterns when these variants are present in patients. Therefore, this study characterized the role of miRNAs harboring variants related to RASopathies for the first time and indicated the possible implications of these variants for phenotypes of RASopathies such as congenital cardiac defects and cardio-cerebrovascular diseases. The expression and existence of miRNA variants may be used in the study of biomarkers of the RASopathies.

## Introduction

RASopathies are a complex and clinically defined group of genetic syndromes caused by germline mutations in genes that encode components or regulators of the RAS/mitogen-activated protein kinase (MAPK) pathway (Rauen, 2013). These mutations are already well described in the following disorders: Neurofibromatosis type I—*NF1* gene; Noonan syndrome (NS)— *PTPN11*, *SOS1*, *RAF1*, *KRAS*, *NRAS*, *SHOC2*, and *CBL* genes; Noonan with multiple lentigines or LEOPARD syndrome—*PTPN11* and *RAF1* genes; Legius syndrome—*SPRED1* gene; Costello syndrome (CS)—*HRAS* gene; Cardiofaciocutaneous syndrome (CFC)—*BRAF* and *MAP2K1* or *MAP2K2* genes; and Capillary malformation-arteriovenous malformation syndrome (CM-AVM)—*RASA1* gene (Tidyman and Rauen, 2016). These syndromes share many overlapping phenotypic features, including craniofacial dysmorphology, cardiac, cutaneous, skeletal and ocular abnormalities, neurocognitive impairment, hypotonia, impaired growth, and in many cases, an increased cancer risk, because of the common underlying Ras/MAPK pathway dysregulation (Aoki et al., 2016; Tidyman and Rauen, 2016).

The advance of the Human Genome Project (HGP) and the rapid development of next-generation sequencing (NGS) technologies have made great advances in precision medicine possible, encompassing the application of modern genetic technologies including molecular and cellular biology analyses at different levels of “OMICS,” such as whole-exome sequencing (WES) (Liu et al., 2019). The WES approach enables studies of the patients within an environmental and clinical context through large-scale cohort trials and data processing with a focus on the associations among molecular biology, disease and health phenotypes to assure a more accurate diagnosis, leading to the establishment of individualized disease prevention and treatment programmes (Tetreault et al., 2015; Collins et al., 2016; Tebani et al., 2016). Moreover, WES has allowed the identification of new genes associated to the clinical description of patients with RASopathies (Niguidula et al., 2018; Matias et al., 2019), such as *RIT1* (Aoki et al., 2013), *A2ML1* (Vissers et al., 2015), *RASA2, SPRY1* (Chen et al., 2014), *SOS2, LZTR1* (Yamamoto et al., 2015; Umeki et al., 2019), *PPP1CB* (Gripp et al., 2016), *CBL* (Coe et al., 2017), and *MRAS* (Higgins et al., 2017), as well as new mutations in genes of the RAS/MAPK pathway (Carapito et al., 2014; Sana et al., 2016; Coe et al., 2017; Harms et al., 2018; Valera et al., 2018).

Although WES is considered an approach for the detection of variants in the exons of protein-coding genes, it also enables the identification of variants in untranslated regions (UTRs) and non-coding regions such as introns, intron-exon boundary regions, and intergenic regions (Guo et al., 2012) as well as non-coding RNA regulators (*e.g.*, small RNAs such as microRNAs and long non-coding RNAs) (Warr et al., 2015). miRNAs can affect the expression of approximately 60% of human genes and are considered the main regulators of gene expression, playing roles in processes such as development and cellular proliferation, consequently triggering diseases (Backes et al., 2016; Nothnick, 2017; Quinlan et al., 2017; Vishnoi and Rani, 2017).

Variants in noncoding DNA sequences such as miRNA genes, especially those located in the “seed” region of mature miRNAs, could disrupt specific miRNA-mRNA target site interactions, resulting in target gene expression dysregulation or affecting the maturation of miRNA (Ghanbari et al., 2017). Indeed, several variants in miRNA genes or target sites have been shown to be associated with human diseases by affecting miRNA-mediated-transcriptional- regulatory function (Kwanhian et al., 2012; Morales et al., 2016; Bastami et al., 2019; Grigelioniene et al., 2019). However, no variants in miRNAs affecting their expression/maturation and consequently dysregulating processes or pathways have yet been described in RASopathy syndromes. Herein, we describe and validate eight miRNA variants from the plasma of five patients previously diagnosed with RASopathies.

## Materials and Methods

### Clinical Data

Five patients (hereafter referred to as P1, P2, P3, P4, and P5) with previous clinical diagnoses of RASopathy syndromes were recruited in 2016 from the National Institute of Women, Children and Adolescents Health Fernandes Figueira (IFF/Fiocruz). The Research Ethics Committee (CEP-IFF/Fiocruz-RJ) approved the study protocol, and all patients or their legal representatives provided written informed consent. This study was carried out in accordance with the guidelines of the Declaration of Helsinki from the World Medical Association. Patients shared at least one of these clinical features such as congenital cardiomyopathy and other heart defects (pulmonary stenosis, ventricular septal defects, valvar pulmonic stenosis), palmar and plantar skin redundancy, prominent fetal pads, hyperlaxity of joints, Noonan phenotype, motor delay, and café-au-lait spots. The study contains four male patients and one female, with ages ranging from 2 to 18 years old. We included samples from two donors (hereafter referred to as C1 and C2) without any family history of RASopathy as control samples in the expression analysis.

### DNA and RNA Extraction and cDNA Synthesis

Genomic DNA (gDNA) was isolated from peripheral blood samples from five patients using the PureLink Genomic DNA Kit (Invitrogen by Thermo Fisher Scientific, Carlsbad, CA, USA) according to the manufacturer’s instructions and stored at −20°C until WES and Sanger sequencing.

Whole peripheral blood samples from the same patients used for WES analysis and two control individuals (without a family history of RASopathy-control individuals) were obtained. Peripheral blood mononuclear cells (PBMC) were extracted by density gradient centrifugation process using Ficoll-Paque. Total RNA was extracted using TRIzol^®^ reagent (Life Technologies, Carlsbad, CA, USA) according to the manufacturer’s instructions. The quantity and quality of the isolated RNA were evaluated using a NanoDrop ND-2000c (Fisher Scientific, Ottawa, Ontario, Canada). The specific stem-loop primer used for miRNA cDNA synthesis was designed as described previously (Chen et al., 2005) (**Supplemental Table 1**). The miRNA cDNA was synthesized by adding 50 ng of total RNA and a specific stem loop primer at 0.5 µM in an initial volume of 10 µl, followed by an elongation step (70°C for 5 min). For the reverse transcription step, 1 U of SuperScript II reverse transcriptase (Life Technologies, Carlsbad, CA, USA); 1X First Strand Buffer, 0.5 mM dNTPs, and 40 U of RNaseOUT Recombinant Ribonuclease Inhibitor (Life Technologies, Carlsbad, CA, USA) were included in a final volume of 20 µl. The cDNA synthesis conditions were 16°C for 30 min, 42°C for 50 min, and 70°C for 15 min.

### Library Construction, Sequencing and Bioinformatic Analysis of Whole-Exome Sequencing

The WES libraries were prepared at the IdenGene Medicina Diagnóstica sequencing facility (São Paulo, Brazil) using a Nextera Rapid Capture Expanded Exome kit (Illumina Inc., San Diego, California, USA) according to the manufacturer’s instructions. Sequencing was performed in paired-end mode with 300 cycles using the NextSeq500/550^®^ Mid Output v2 kit (Illumina Inc., San Diego, California) in a NextSeq500 sequencer (Illumina Inc., San Diego, California, USA).

The bioinformatic analysis was performed at LABINFO/LNCC (Petrópolis, Rio de Janeiro, Brazil), including quality evaluation of the WES libraries using the FASTQC tool (http://www.bioinformatics.babraham.ac.uk/projects/fastqc/). The removal of reads or fragments with low quality was performed with Trimmomatic software (http://www.usadellab.org/cms/?page=trimmomatic). The resulting high-quality reads were mapped to the reference human genome (version GRCh38) using Bowtie2 (Langmead and Salzberg, 2012) with a very sensitive presetting allowing one mismatch per seed region. Optical duplicates were marked by Picard 2.18 (http://broadinstitute.github.io/picard/). Variant calling was performed with the Genome Analysis Toolkit (GATK) version 3.8 (McKenna et al., 2010) using HaplotypeCaller with variant quality score recalibration (DePristo et al., 2011; Van der Auwera et al., 2013). The calling variants with read coverage of ≤5 reads and MAP quality (MAPQ) ≤ 30 were filtered out to avoid false positives. The SnpEff/SnpSift (Cingolani et al., 2012a; Cingolani et al., 2012b) version 4.3r tools were used to predict and annotate the functional impact of variants along with the dbSNP (build 151) and dbNSFP version 3.5 databases (Sherry et al., 2001; Liu et al., 2011).

After the variant annotation step, we retrieved the miRNA *loci* harboring rare variants, *i.e.* those with a 5% Minor Allele Frequency (MAF) in at least one populational frequency database such as the 1,000 Genomes Project (Lek et al., 2016), 6,500 NHLBI GO Exome Sequencing Project (Tennessen et al., 2012), Exome Aggregation Consortium (Lek et al., 2016), TOPMed (NHLBI Trans-Omics for Precision Medicine WGS-About TOPMed) and ABraOM (Naslavsky et al., 2017) databases. The variants in mature miRNAs associated with known phenotypes of RASopathies as well as cardio-cerebrovascular diseases (Calcagni et al., 2017; Chacon-Camacho et al., 2019); (Cao et al., 2017) and cancer diseases (Hernández-Martín and Torrelo, 2011; Villani et al., 2017; Keshari et al., 2018; Rauen et al., 2018) were also selected. All known predicted and validated target genes from the polymorphic miRNA list were retrieved from the DIANA tools online suite (Vlachos and Hatzigeorgiou, 2017) and miRTARBASE with the SpiderMiR package (Cava et al., 2017) in R. The resulting list of target genes was used as a query for over-representation analysis with the KEGG and Gene Ontology (GO) databases using the clusterProfiler package (Yu, 2018) in R. Over-representation analysis of rare diseases according to the NIH (National Institutes of Health) and GARD (Genetic and Rare Disesases Information Center) was performed using the ARCHS4 predictions of GeneRIF with the Enrichr web tool (Kuleshov et al., 2016; Lachmann et al., 2018). The target genes of the miRNAs related to RAS/MAPK/PI3K-Akt pathways or phenotypes associated with RASopathy syndromes were retrieved for posterior analysis. The effect of the variants on the secondary structure of miRNA precursors was evaluated with the MFOLD web tool (Zuker, 2003) using the RNA folding form to generate secondary structures with and without variants. Additional criteria for miRNA variants prioritization were association studies with cardiovascular diseases, cardiac abnormalities, and cancer regardless of MAF value, as found in previous studies (Hernández-Martín and Torrelo, 2011; Cao et al., 2017; Calcagni et al., 2017; Villani et al., 2017; Keshari et al., 2018; Rauen et al., 2018; Chacon-Camacho et al., 2019).

### Confirmation of Variants in miRNAs by Sanger Sequencing

The selected variants in mature miRNAs were confirmed by Sanger sequencing. Briefly, the gDNA from the five patients was amplified by PCR with specific primers (**Supplemental Table 1**). The amplicons were purified using a PureLink PCR purification kit (Life Technologies, Carlsbad, CA, USA) and sequenced on an ABI 3730 automated DNA sequencer (Applied Biosystems by Thermo Fisher Scientific, Carlsbad, CA, USA) using BigDye sequencing buffer (Life Technologies, Carlsbad, CA, US). The resulting chromatograms were analyzed with the Gentle tool (http://gentle.magnusmanske.de/).

### miRNA Expression Analysis by RT-qPCR

The RT-qPCR data were generated with three RNA replicates for each patient and individual control. RT-qPCR was performed using a StepOnePlus System (Applied Biosystems by Thermo Fisher Scientific, Carlsbad, CA, USA) with PowerUp SYBR Green Master Mix (Life Technologies, Carlsbad, CA, USA). The primer sequences used for RT-qPCR analysis are shown in **Supplemental Table 1**. The amplification data and results for each plate were exported from the StepOnePlus System in a CSV spreadsheet file for posterior statistical analysis.

### Statistical Analysis

The RT-qPCR amplification data (Rn data) were used to fit four- to seven-parameter logistics with the *qpcR* library in the R language (Ritz and Spiess, 2008), retrieving the PCR efficiency and Cq from the sigmoidal fit of the best-performing model. hsa-miR-16-5p was used as a normalization control (Lange et al., 2017; Zalewski et al., 2017; Rinnerthaler et al., 2016). The expression analysis of the miRNAs was carried out using the model with correction of the PCR efficiency (Pfaffl, 2002), where the relative expression ratio of a target miRNA is calculated based on its real-time PCR efficiency (E) and the crossing point (CP or Cq) difference between the five patients with RASopathies and the two healthy individuals (controls). Statistical analysis and plotting were performed in R. The Shapiro-Wilk and Levene tests were applied to evaluate data normality and homogeneity, respectively. We used one-way ANOVA followed by Tukey’s test with a significance level was set at 5%.

## Results

### Identification of miRNA Variants Related to RASopathies

**Figure 1:**
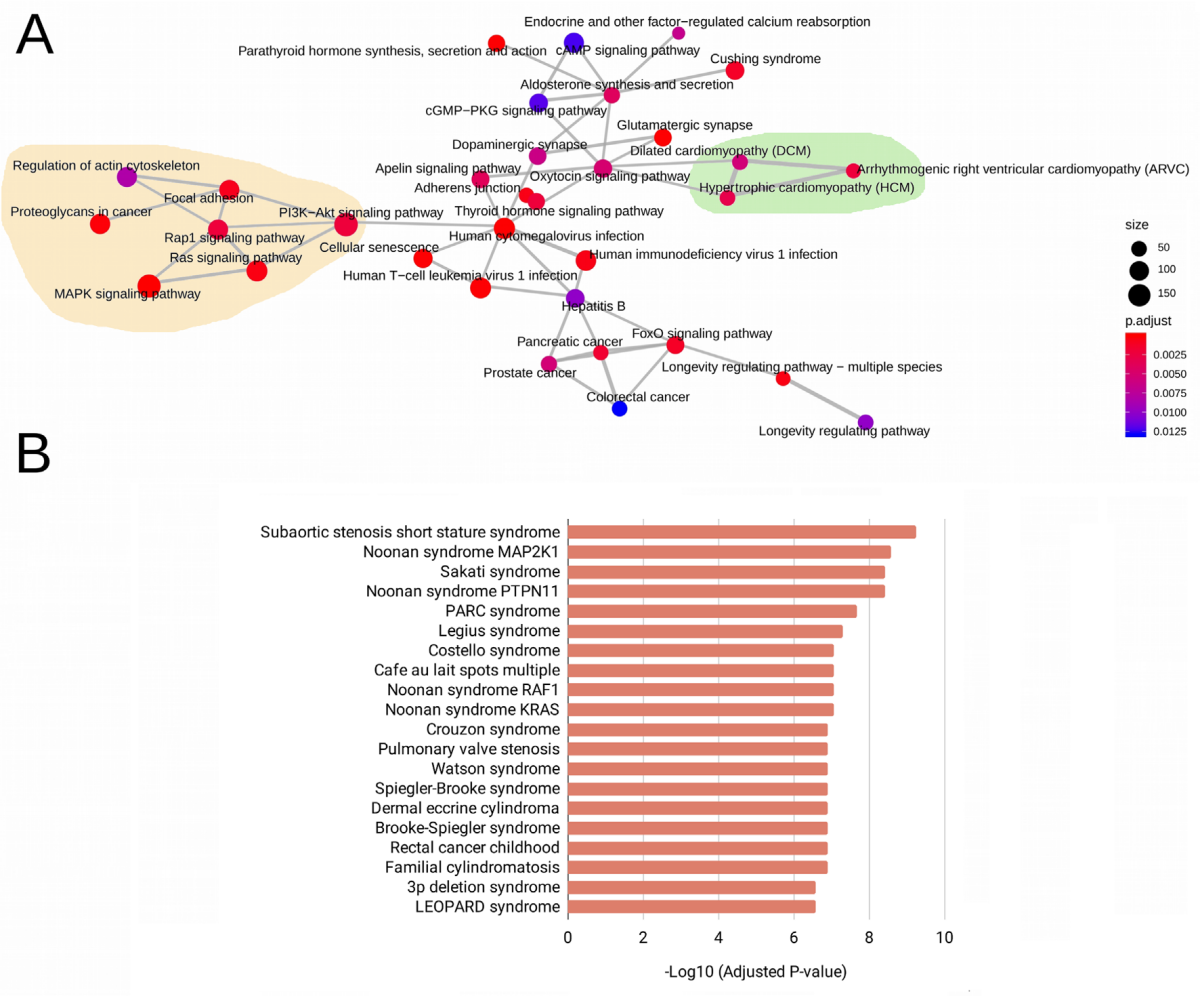
Enriched pathways obtained from KEGG terms. **(A)** Rare disease terms from the National Institutes of Health (NIH) **(B)** for the target genes of 41 evaluated mature miRNAs. In **(A)**, the size of the circle represents the number of genes involved in the metabolic pathway. The color intensity scale represents the statistical significance (adjusted *P*-value) of the enriched KEGG pathways generated by clusterProfiler. Pale yellow areas represent enriched RAS/MAPK and PI3K-Akt pathways; green areas represent enriched diseases such as hypertrophic and dilated cardiomyopathies. In **(B)**, the bars represent the statistical significance (adjusted *P*-values) of the enriched rare diseases generated from the ARCHS4 predictions of GeneRIF in Enrichr.

The WES analysis of the five patients with RASopathies revealed 41 variants in mature miRNAs distributed among patients (**Supplemental Table 2** and **Data Sheet 2**). The experimentally validated and predicted target genes of the 41 polymorphic miRNAs were recovered and used for gene set enrichment analysis with the KEGG, GO (clusterProfiler) and rare disease (Enrichr) databases to identify which pathways, biological processes, and rare diseases, respectively, might be regulated by the miRNAs. This analysis indicated pathways, biological processes, and diseases associated with RASopathies that were enriched, which included the RAS, MAPK, RAP1, and PIK3-Akt signaling pathways and proteoglycans in cancer as well as congenital cardiac defects such as hypertrophic cardiomyopathy (HCM), dilated cardiomyopathy (DCM), and arrhythmogenic right ventricular cardiomyopathy (ARVC) as shown in **Figure 1A**. Surprisingly, these target genes were enriched in rare diseases such as subaortic stenosis short stature syndrome, Noonan syndrome, Legius syndrome, Costello syndrome, cafe au lait spots multiple, pulmonary valve stenosis, and LEOPARD syndrome (**Figure 1B**). The enriched GO biological process (BP) terms included negative regulation of neuron differentiation, neurogenesis, nervous system and cell development, axonogenesis, RAS protein signal transduction, regulation of Wnt signaling pathway, and cell-cell signaling by Wnt (**Supplemental Figure 1A**).

**Figure 2:**
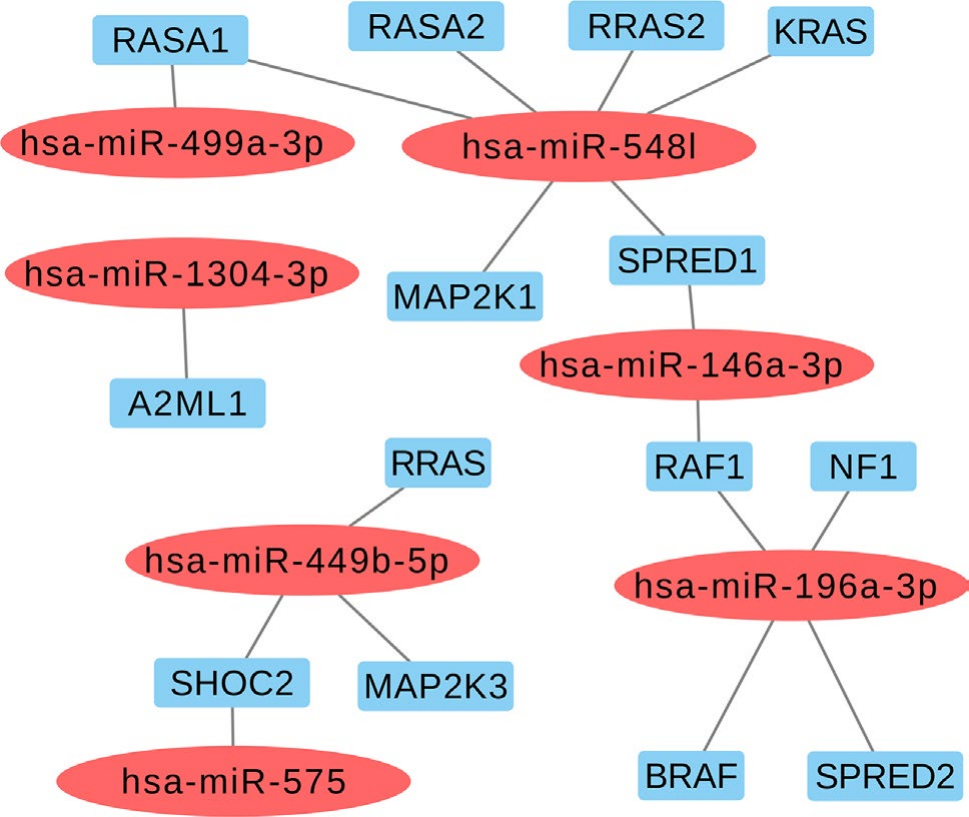
Selected miRNAs based in regulating of the classical genes from RAS-MAPK pathways involved with RASopathies.

Based on the aforementioned analysis, we selected nine miRNAs (hsa-miR-1304-3p, hsa-miR-146a-3p, hsa-miR-196a-2-3p, hsa-miR-499a-3p, hsa-miR-499b-5p, hsa-miR-449b-5p, hsa-miR-548l, hsa-miR-575, and hsa-miR-593-5p) and eight variants located in these mature miRNAs. Furthermore, we found that six selected miRNAs in this study may also act as regulators of classical genes involved with RASopathies, such as hsa-miR-146a-3p (*SPRED1* and *RAF1*), hsa-miR-196a-3p (*RAF1, NF1, BRAF,* and *SPRED2*), hsa-miR-548l (*RASA1, RASA2, RRAS2, KRAS, MAP2K1,* and *SPRED1*), hsa-miR-449b-5p (*RRAS, SHOC2,* and *MAP2K3*), hsa-miR-575 (*SHOC2*), and hsa-miR499a-3p (*RASA1*) as shown in **Figure 2** and **Supplemental Table 3**.

### miRNA Variants and Modifications in the Secondary Structure of MicroRNA Precursors

Among the eight variants found in the miRNAs, two were homozygous, which were identified in hsa-miR-1304-3p (rs2155248, NR_031639.1:n.65 C > A) in patients P2, P3, P4, and P5 and hsa-miR-146a-3p (rs2910164, NR_029701.1:n.60C > G) in all patients (**Table 1**). Five heterozygous variants were identified in single patients, which were located in hsa-miR196a2-3p (rs11614913, NR_029617.1:n.78C > T) in P3, hsa-miR-548l (rs13447640, NR_031630.1:n.26C > T) in P5, hsa-miR-449b-5p (rs10061133, NR_030387.1:n.27T > C) in P1, miR-575 (rs149186367, NR_030301.1:n.64C > T) in P4, and hsa-miR-593-5p (rs73721294, NR_030324.1:n.22C > T) in P1. The eighth variant was heterozygous and was found in two miRNAs encoded from opposite strands of the same locus: hsa-miR-499a-3p on the plus strand (rs3746444, NR_030223.1:n.73A > G) and hsa-miR-499b-5p on the minus strand (reverse) (rs3746444, NR_039912.1:n.25T > C) within the genomes of patients P1, P3, and P5 (**Table 1**).

**Table 1:** Variants in mature miRNAs found in patients with RASopathy syndromes.

MicroRNA Acronym	SNP ID	Minimum Allele Frequency (MAF)	Position (chromosome:position)	Nucleotide change	Zygosity in Patients (P1-P5)
hsa-miR-1304-3p	rs2155248	G = 0.0399 (ExAC) G = 0.1208 (1,000 Genomes) G = 0.0978 (GO-ESP) G = 0.925287 (AbraOM)	11:93733700	G > T (REV C > A)	P2, P3, P4, and P5 are Homozygous
hsa-miR-146a-3p	rs2910164	C = 0.2792 (ExAC) C = 0.2797 (GO-ESP), C = 0.703612 (AbraOM)	5:160485411	C > G	P1-P5 are Homozygous
hsa-miR-196a2-3p	rs11614913	T = 0.4220 (ExAC) T = 0.3327 (1,000 Genomes) T = 0.3406 (GO-ESP) T = 0.336617 (AbraOM)	12:53991815	C > T	P3 is Heterozygous
hsa-miR-499a-3p	rs3746444	G = 0.2013 (ExAC) G = 0.1835 (1,000 Genomes) G = 0.1951 (GO-ESP) G = 0.192118 (AbraOM)	20:34990448	A > G	P1, P3, and P5 are Heterozygous
hsa-miR-499b-5p	rs3746444	G = 0.2013 (ExAC) G = 0.1835 (1,000 Genomes) G = 0.1951 (GO-ESP) G = 0.192118 (AbraOM)	20:34990448	A > G (REV T > C)	P1, P3, and P5 are Heterozygous
hsa-miR-548l	rs13447640	A = 0.0325 (ExAC) A = 0.0519 (1,000 Genomes) A = 0.0494 (GO-ESP)	11:94466555	G > A (REV C > T)	P5 is Heterozygous
hsa-miR-449b-5p	rs10061133	G = 0.1111 (ExAC) G = 0.1222 (1,000 Genomes) G = 0.0851 (GO-ESP) G = 0.085386 (AbraOM)	5:55170716	A > G (REV T > C)	P1 is Heterozygous
hsa-miR-575	rs149186367	A = 0.0007 (ExAC) A = 0.0026 (1,000 Genomes) A = 0.0019 (GO-ESP) A = 0.000821 (AbraOM)	4:82753367	G > A (REV C > T)	P4 is Heterozygous
hsa-miR-593-5p	rs73721294	T = 0.0063 (ExAC) T = 0.0270 (1,000 Genomes) T = 0.0168 (GO-ESP) T = 0.009852 (AbraOM)	7:128081882	C > T	P1 is Heterozygous

The results obtained in the patients suggest the involvement of a combination of variants, where P1 (hsa-miR-146a-3p-GG, hsa-miR-499a-3p-AG/hsa-miR-499b-5p-TC, hsa-miR-449b-5p-TC, and hsa-miR-593-5p-CT), P3 (hsa-miR-1304-3p-AA, hsa-miR-146a-3p-GG, hsa-196a-2-3p-CT, and hsa-miR-499a-3p-AG/hsa-miR-499b-5p-TC), and P5 (hsa-miR-1304-3p-AA, hsa-miR-146a-3p-GG, hsa-196a-2-3p-CT, and hsa-miR-499a-3p-AG/hsa-miR-499b-5p-TC) all carried five variants, while P4 (hsa-miR-1304-3p-AA, hsa-miR-146a-3p-GG and hsa-miR-575-CT) and P2 (hsa-miR-1304-3p-AA, hsa-miR-146a-3p-GG) carried three and two variants, respectively. Studies involving a larger cohort could evaluate possible synergic effects of these variant combinations associated with RASopathy syndromes.

**Figure 3:**
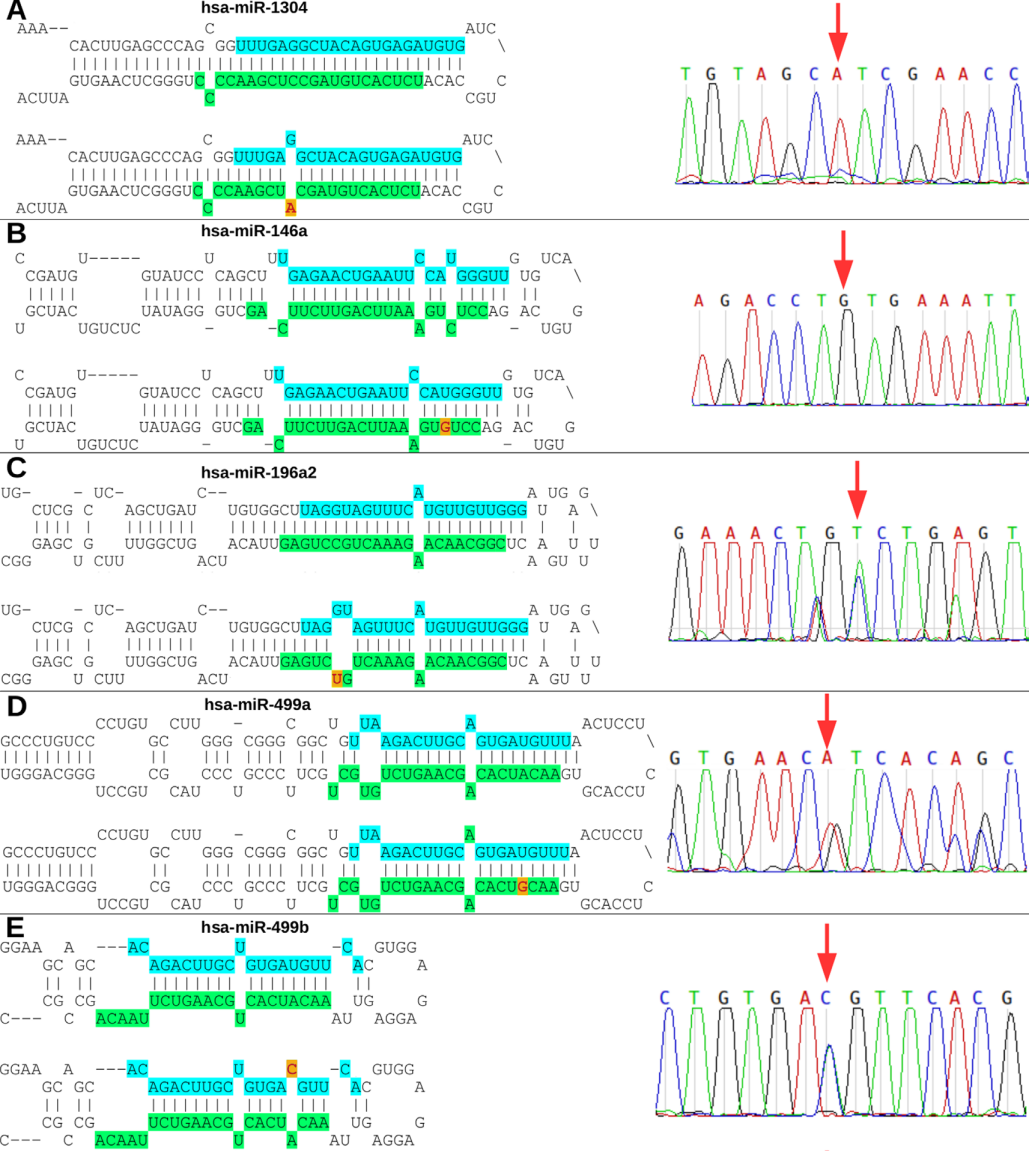
Nucleotide modifications in miRNA precursors induced by variants. **(A)** The rs2155248 variant results in a C A change in hsa-miR-1304, altering G-C pairing to G-A pairing in patient P2 (homozygous genotype AA); **(B)** variant rs2910164 results in a C G change in hsa-miR-146a, altering U-C pairing to U-G pairing in patient P1 (homozygous genotype GG); **(C)** variant rs11614913 results in a C T change in hsa-miR-196a2, altering G-C pairing to G-U pairing in patient P3 (heterozygous genotype CT); **(D)** variant rs3746444 results in a A G change in hsa-miR-499a, altering U-A pairing to U-G pairing in patient P5 (heterozygous genotype AG); and **(E)** variant rs3746444 results in a T C change in hsa-miR-499b, altering U-A pairing to C-A pairing in patient P5 (heterozygous genotype TC). Blue and green in the secondary structure represent the position in the mature miRNA on the 5’ (5p) and 3’ (3p) arms, respectively. The red letter with a yellow background represents the variant described in the study. The chromatograms of each miRNA with a variant are also shown.

The rs2155248 G65T variant (reverse strand C65A) is located at the 65th position of hsa-miR-1304 (13th position of miR-1304-3p). This variant modifies base-pairing C-G base pairs to A-G base pairs, causing a mismatch in the secondary structure (**Figure 3A**). The rs2910164 variant (C > G) is located at the 60th position of hsa-miR-146a in the seed region, and induces wobble pairing (G-U) between hsa-miR-146a-3p and hsa-miR-146a-5p (**Figure 3B**). The rs11614913 variant (C > T) is located at the 78th position of hsa-miR-196a2 (17th base) in the out-seed region, and induces wobble pairing (**Figure 3C**). The rs3746444 variant (A > G) is located at the hsa-miR-499a and hsa-miR499b *loci* (**Figures 3D, E**) in the 73rd position of hsa-miR-499a (4th position of hsa-miR-499a-3p) and at the 25th position of hsa-miR-499b (16th position of miR-499b-5p). Therefore, it is referred to as the hsa-miR-499 A73G variant and induces G-U wobble pairing in miR-499a and a C-A mismatch in miR-499b in the seed regions of these miRNAs.

The rs13447640 G > A variant (reverse strand C > T) is located in hsa-miR-548l in the 26th position of the 5’ arm, representing the 12th position in hsa-miR-548l-5p. This variant induces U-G wobble pairing (**Supplemental Figure 2A**). The rs10061133 A > G variant (reverse strand T > C) is located in hsa-miR-449b at the 27th position of the 5’ arm (12th position of hsa-miR-449b-5p). The variant introduces a gap in the secondary structure of hsa-miR-449b (**Supplemental Figure 2B**). The variant rs149186367 G > A (reverse strand C > T) is located in hsa-miR-575 in the 64th position of the 3’ arm (4th position in the seed region of hsa-miR-575-3p). This variant induces G-U wobble pairing (**Supplemental Figure 2C**). The rs73721294 variant (C > T) is located in hsa-miR-593 in the 22nd position of the 5’ arm (7th position in the seed region of hsa-miR-593-5p). The variant introduces a U-U mismatch in hsa-miR-593-5p (**Supplemental Figure 2D**).

### miRNA Expression Analysis

**Figure 4:**
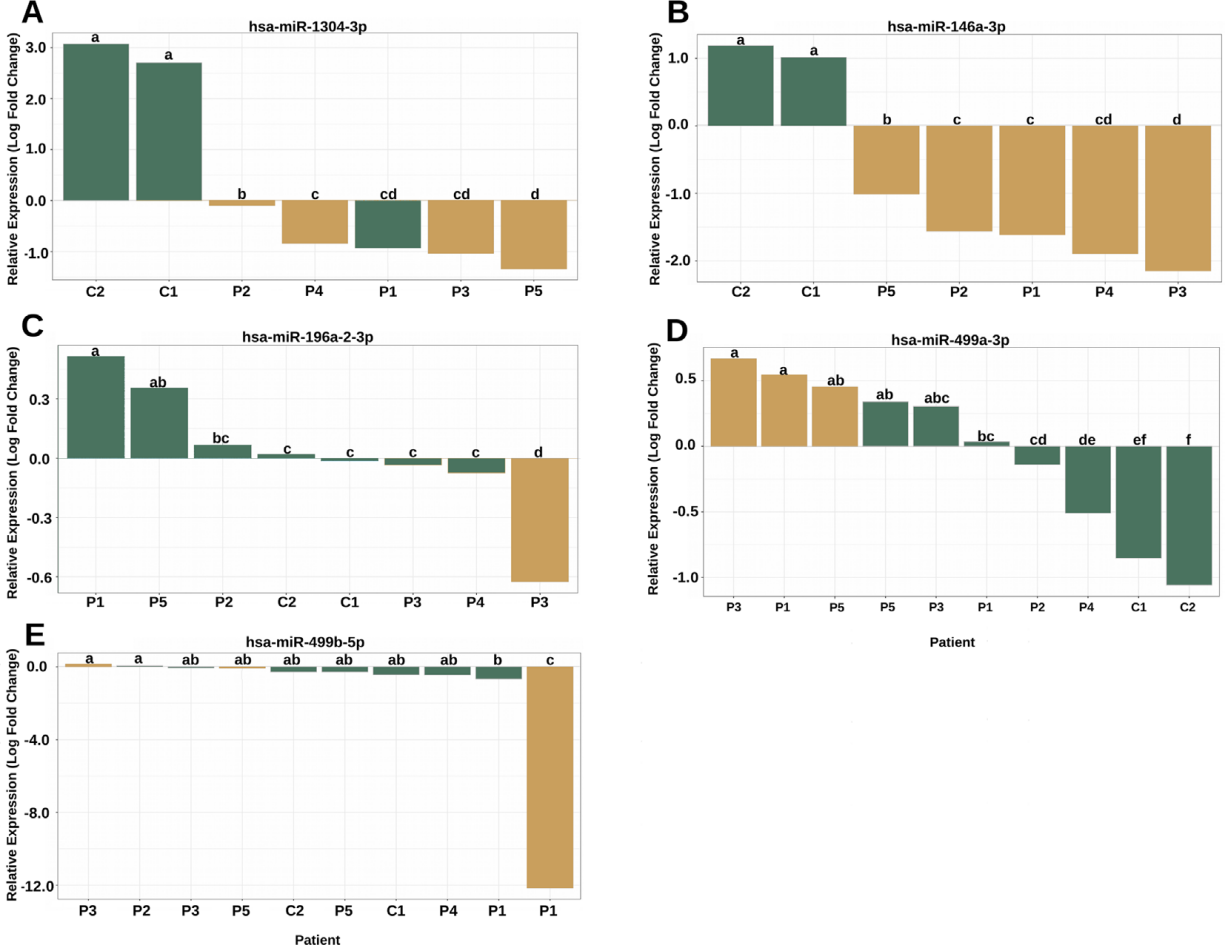
The variants affect the expression of miRNAs in patients with RASopathies. **(A)** Homozygous rs2155248 variant (G T-rev C A) in hsa-miR-1304-3p in patients P2, P3, P4, and P5, genotyped as AA; **(B)** homozygous rs2155248 variant (C G) in hsa-miR-146a-3p, found in all patients genotyped as GG; **(C)** heterozygous rs11614913 variant (C T) in hsa-miR-196a-2-3p in patient P3, genotyped as CT; **(D)** heterozygous rs3746444 variant (A G) in hsa-miR-499a-3p in patients P1, P3, and P5, genotyped as AG; **(E)** heterozygous rs3746444 variant (T C) in hsa-miR-499b-5p in patients P1, P3, and P5 genotyped as TC. The other referenced patients were genotyped as homozygous for the reference allele of the variant. The yellow and green bars represent the relative expression of miRNAs with and without variants, respectively. Means with the same lowercase letter are not significantly different from each other by Tukey test (P > 0.05).

The analysis of the secondary structure of the miRNAs motivated us to further study whether the variants could affect the plasma levels of miRNAs and the clinical features of patients. The expression analysis revealed four miRNAs with altered expression profiles compared to the controls (**Figure 4**). hsa-miR-1304-3p was downregulated in all patients, including P1, who did not harbor the variant and presented expression of the reference allele (**Figure 4A**). It was also observed that the level of polymorphic miRNA expression was different among four patients (P2, P4, P3, and P5). hsa-miR-146a-3p harboring a variant exhibited downregulation in all patients (**Figure 4B**) and showed differences between patients diagnosed with Rasopathies syndromes. The expression of the variant allele of hsa-miR-196a2-3p in P3 was significantly decreased in comparison with the reference allele, controls and other patients (**Figure 4C**), which demonstrates that the variant could induce downregulation of miRNA in this patient. hsa-miR-499a-3p harboring a variant was upregulated in P1, P3, and P5 compared to the other patients and controls (**Figure 4D**). hsa-miR-499b-5p containing a variant showed a strong reduction in expression in P1 compared to the reference allele, other patients and controls (**Figure 4E**).

hsa-miR-548l, hsa-miR-449b-5p, hsa-miR-575 and hsa-miR-593-5p harboring variants showed only a slight difference in expression compared with the controls and/or the reference allele (**Supplemental Figure 3**). In summary, the miRNA expression analysis showed that the levels of the miRNAs in blood could be influenced by the presence of genetic variants.

## Discussion

miRNAs have attracted global attention due to their role as transcriptional regulators in different cell types, developmental processes and the pathogenesis of diseases. Variants located in miRNAs may directly affect miRNA maturation, expression, and binding to target mRNAs and consequently alter the expression levels of target genes-miRNAs affecting the signaling pathways related to the progression and prognosis of diseases. In the present study, we showed that miRNAs harboring variants may regulate genes involved in the RAS-MAPK and PI3K-AKT cascades, which are the main pathways involved in RASopathies (Rauen, 2013; Tidyman and Rauen, 2016; Zhong, 2016). We also showed that these miRNAs can regulate genes related to RASopathies-associated phenotypes such as hypertrophic cardiomyopathy (HCM), subaortic stenosis, pulmonary valve stenosis, and cafe-au-lait spots, some of which are observed in the patients evaluated in the present study.

Neuronal differentiation, neurogenesis, nervous system and cell development and axonogenesis were also enriched biological processes identified in our analyses, corroborating the data on the RAS-MAPK-PI3K-AKT pathways. It is known that these pathways play an essential role in proliferation, differentiation, migration, and developmental processes. Moreover, in the group of patients diagnosed with RASopathies by this work was observed phenotypic abnormalities in the central nervous system such as developmental delay, macrocephaly, neurocognitive deficits, seizures or structural malformations confirming results of enrichment analysis of KEGG and GO from target genes. These results corroborate other studies demonstrating that these phenotypes are associated with RASopathies (Zhong, 2016; Kim and Baek, 2019). Furthermore, we found that miRNAs in this study may also act as regulators of classical genes involved with RASopathies and this could be indirectly interfering in phenotypes of patients as shown in other diseases (Cammaerts et al., 2015; Ghanbari et al., 2017). However, more studies are required to validate these results obtained *in silico*.

In our study, the miRNAs harboring variants showed remarkable alterations in their secondary structures, and this may have affected the expression profile of miRNAs in the plasma of patients with RASopathies. This fact has already been observed in selected miRNA variants, including processing and/or target recognition by miRNAs in rs3746444 located within the pre-miR-499, affecting the maturing of miR-499-5p, and interfering in the antiapoptotic function by converting stable A-U base pair to wobble G-U base pair in pre-miR-499 secondary structure (Ding et al., 2018). Another study showed that rs11614913 in the miR-196a2 sequence could alter mature expression and target mRNA binding (Xu et al., 2009). Moreover, some studies have suggested that an alteration of base pairing in the secondary structure is enough to affect miRNA maturation, processing by Drosha and Dicer, and duplex stability (Ding et al., 2013; Ding et al., 2018).

We observed downregulation of miRNA-146a-3p, miR-1304-3p, miR-196a2-3p, and miR-499b-5p and upregulation of miR-499a-3p in patients harboring the variants. Patients P1 and P3 presented the rs3746444 variant in hsa-miR-499a/b, resulting in an altered expression profile with common phenotypic features of RASopathies, congenital cardiomyopathy, and pulmonary stenosis. These data corroborate the results found in other studies that have suggested cardiovascular diseases are associated with this miRNA variant (Fawzy et al., 2018; Li et al., 2018). The miRNA hsa-miR-499 has previously been associated with the development and functioning of the heart, showing high expression in cardiac tissue, and with alterations in physiological and pathological processes during myocardial injury and remodelling (Dorn et al., 2012). Other variants such as miR-146a rs2910146 and miR-196a2 rs11614913 may also affect the phenotypes of these patients, since these variants have been described as being associated with cardiovascular diseases (Xu et al., 2009; Ramkaran et al., 2014; Xiong et al., 2014; Chen et al., 2017; Liu et al., 2017; Guo et al., 2018; Bastami et al., 2019).

Moreover, we found possible combinations of variants in miRNAs (hsa-miR-1304-3p-hsa-miR146a-3p-hsa-miR-196a2-3p-hsa-miR-499a-3p/hsa-miR-499b-5p) between the patients with RASopathies, in accord with the findings of Sung et al. (2016). These authors showed that variants in miRNAs act in specific combinations of haplotypes, resulting in a probable synergistic effect on the incidence of coronary artery disease (CAD). Although no association studies have been achieved due to the low number of patients included in this preliminary study, data suggest a possible effect of combined haplotypes on patients, resulting in similar phenotypes between different syndromes such as cardiomyopathy.

Therefore, our *in silico* analysis also allowed us to propose that the miRNA variants could play indirect roles in RASopathies, regulating genes in canonical pathways and consequently generating different phenotypes exhibited by the patients. Although the association of pathogenic variants or mutations in classical genes of the RAS/MAPK pathways with RASopathies is already well studied (Aoki et al., 2016; Chacon-Camacho et al., 2019), there has been no reported study showing the effects of variants in miRNAs in RASopathies. This is the first study to reveal variants in miRNAs with altered expression profiles in patients diagnosed with RASopathies.

Previous studies have shown the expression profiles of miRNAs in Neurofibromatosis type 1 (Masliah-Planchon et al., 2013; Presneau et al., 2013), Costello syndrome (García-Cruz et al., 2015) and Noonan Syndrome/juvenile myelomonocytic leukaemia (Mulero-Navarro et al., 2015), but these studies did not characterize variants in miRNAs and their possible effect on the expression profile of patients. Thus, although mutations in classical genes of the RAS/MAPK pathway already are associated with RASopathy syndromes, there have been no studies examining non-coding RNAs (miRNAs) related to these syndromes, which shows the relevance of studies on variants located in gene expression regulators such as miRNAs that may contribute to disease development and severity.

The present work described a possible contribution of miRNA variants to the congenital heart defects and cardiomyopathy presented by patients with RASopathy syndromes. Moreover, we showed for the first time that some common phenotypes of RASopathies may also be caused by miRNA dysfunctions. Future studies could use these variants as well as the expression pattern of miRNAs to study molecular markers of these syndromes, which could contribute to improving the diagnosis of these diseases.

## Data Availability Statement

All datasets of WES generated and analyzed (raw data of miRNA variants) for this study are included in the article/Supplementary Material.

## Ethics Statement

The studies involving human participants were reviewed and approved by Research Ethics Committee-CEP-IFF from National Institute of Women, Children and Adolescents Health Fernandes Figueira (IFF/Fiocruz, Rio de Janeiro, Brazil). Written informed consent to participate in this study was provided by the participants’ legal guardian/next of kin.

## Author Contributions

JC, GM, and AV contributed to conception and design of the study. JC and GM performed the statistical analysis of RT-qPCR data, WES, and Sanger Sequencing analyses. JC wrote the draft of the manuscript. TV, NR, JL, SG, GM, and AV contributed with reagents and biological samples, Sanger Sequencing, and critical evaluation of the manuscript. All authors contributed to manuscript revision, read and approved the submitted version.

## Funding

This study was supported by grants from the Fundação de Amparo à Pesquisa do Estado do Rio de Janeiro—FAPERJ # E-26/202.826/2018(BR), Conselho Nacional de Desenvolvimento Científico e Tecnológico–CNPq #303170/2017-4 (BR), and Coordenação de Aperfeiçoamento de Pessoal de Nível Superior (CAPES) [Biocomputational Process Grant number 23038.010041/2013¬13].

## Conflict of Interest

The authors declare that the research was conducted in the absence of any commercial or financial relationships that could be generated a potential conflict of interest.
