# Supplementary material for: miRNA Genetic Variants Alter Their Secondary Structure and Expression in Patients With RASopathies Syndromes
Source: Front Genet. 2019 Nov 13;10:1144. doi: 10.3389/fgene.2019.01144 (PMC6863982; doi:10.3389/fgene.2019.01144)
Supplement: Supplementary file 1 [file DataSheet_1.pdf]

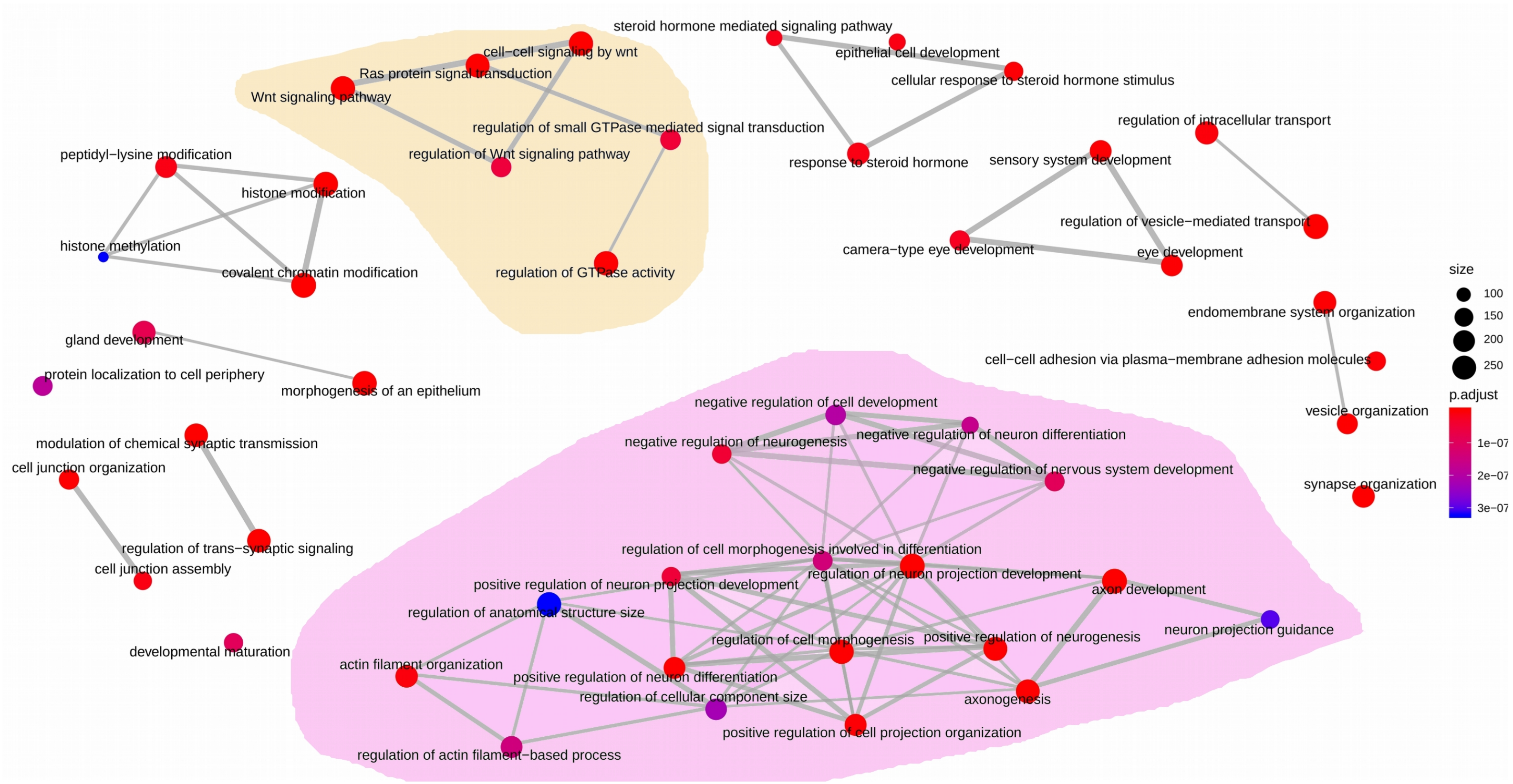

Supplementary Figure 1. Enriched biological process (BP) terms from the Gene Ontology (GO) analysis of the target genes of 41 evaluated mature miRNAs. The size of the circle represents the number of genes involved in the biological process. The colour intensity scale represents the statistical significance (adjusted P-values) of the enriched BP terms generated by clusterProfiler tool. Pale yellow areas represent enriched BP terms related to the RAS and Wnt signalling pathways. The pink area represents enriched BP terms related to neuronal development.

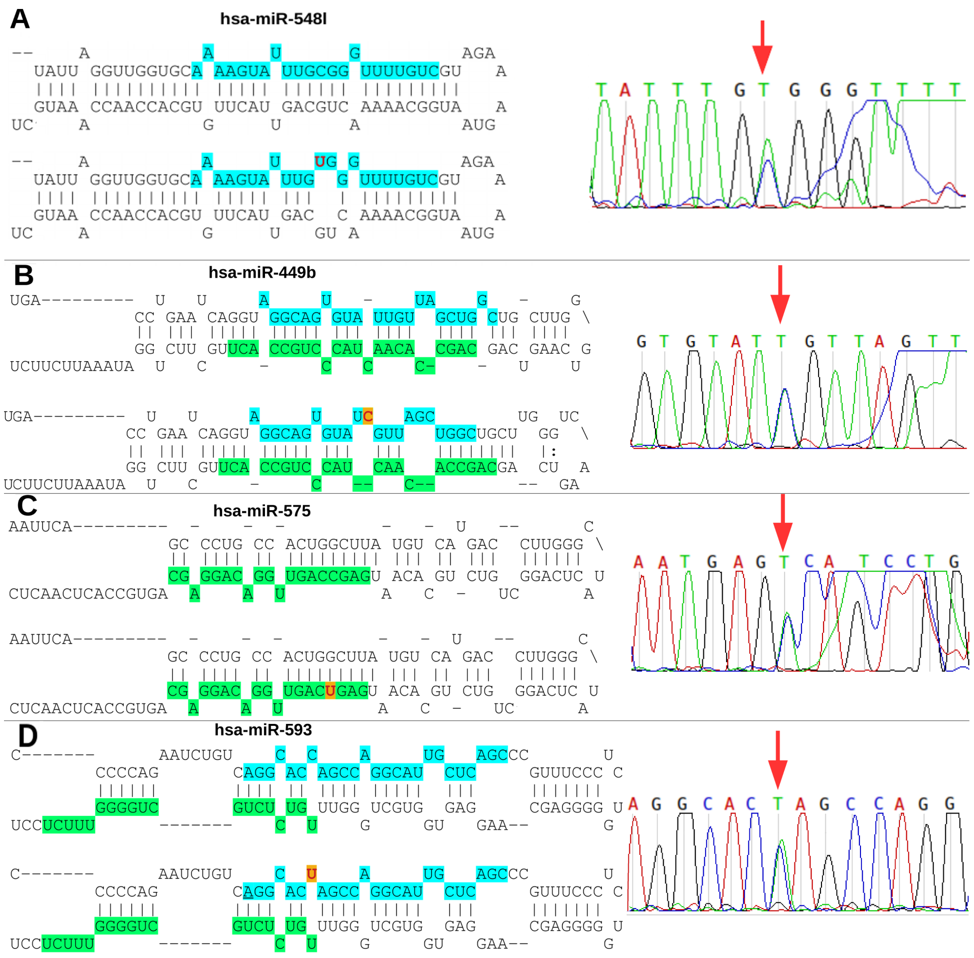

Supplementary Figure 2. The nucleotide modifications in miRNA precursors induced by variants. (**A**) The rs13447640 variant results in a G>A change (REV C>T) in hsa-miR-548l from patient P5 (heterozygous genotype CT); (**B**) the rs10061133 variant results in a A>G change (REV T>C) in hsa-miR-449b-5p, altering pairing in patient P1 (heterozygous genotype TC); (**C**) The rs149186367 variant results in a G>A change in hsa-miR-575, altering pairing in patient P4 (heterozygous genotype GA); (**D**) the rs73721294 variant results in a C>T change in hsa-miR-593-5p, altering pairing in patient P1 (heterozygous genotype CT). Blue and green in the secondary structure represent the positions in the mature miRNA on the 5' (5p) and 3' (3p) arms, respectively. The red letter with a yellow background represents the variant described in the study. The chromatograms of each miRNA with a variant are also shown.

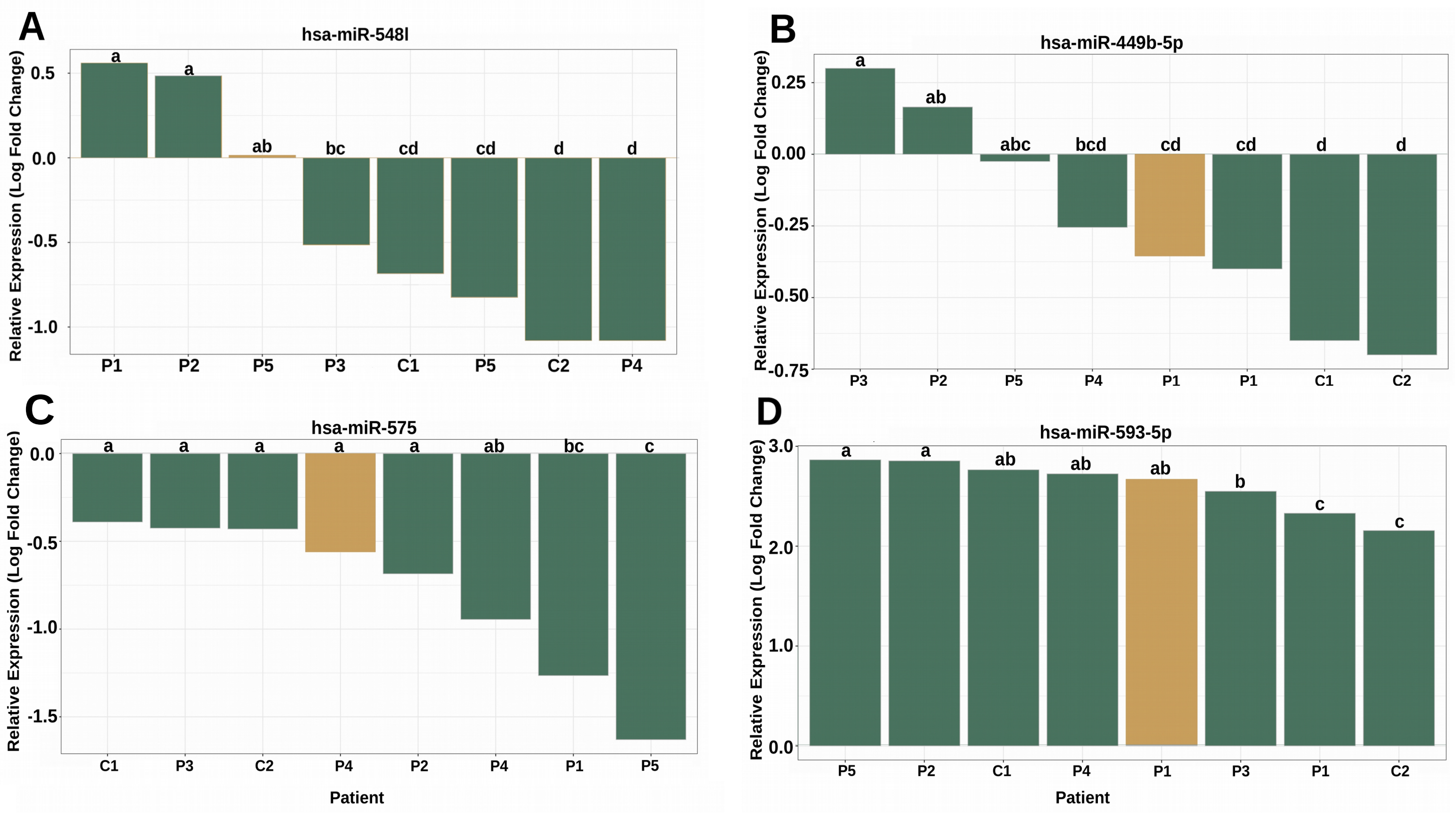

Supplementary Figure 3. The identified variants affect the expression of miRNAs in patients with RASopathies. (**A**) Heterozygous rs13447640 variant (G>A - rev C>T) in hsa-miR-548l in P5, genotyped as CT; (**B**) heterozygous rs2155248 variant (A>G - rev C>T) in hsa-miR-449b-5p in P1, genotyped as CT; (**C**) heterozygous rs149186367 variant (G>A) in hsa-miR-575 in P4, genotyped as GA; (**D**) heterozygous rs73721294 variant (C>T) in hsa-miR-593-5p in P1, genotyped as CT. The other reference patients were genotyped as homozygous for the reference allele of the variant. The yellow and green bars represent the relative expression of miRNAs with and without variants, respectively.
