# Supplementary material for: miRNA Genetic Variants Alter Their Secondary Structure and Expression in Patients With RASopathies Syndromes
Source: Front Genet. 2019 Nov 13;10:1144. doi: 10.3389/fgene.2019.01144 (PMC6863982; doi:10.3389/fgene.2019.01144)
Supplement: Supplementary file 3 [file Table_1.docx]

**Supplementary Table 1.** Primers used in Sanger sequencing, miRNA cDNA synthesis and miRNA expression

| **name** | **Sequence 5’-3’** | **Use** |
| --- | --- | --- |
| miR575_F | CAGAGCCAGTTGGACAGGAGC | miRNA expression |
| miR575_M_F | GCAGAGtCAGTTGGACAGGAGC | miRNA expression |
| miR575_SL | gtcgtatccagtgcagggtccgaggtattcgcactggatacgacGCTCCT | miRNA cDNA synthesis |
| miR449b-5p_F | GCAAGGCAGTGTATTGTTAGCTGGC | miRNA expression |
| miR449b-M_F | CAAGGCAGTGTATcGTTAGCTGGC | miRNA expression |
| miR449b-5p_SL | gtcgtatccagtgcagggtccgaggtattcgcactggatacgacGCCAGC | miRNA cDNA synthesis |
| miR146a-3p_F | TCAGGCACCTCTGAAATTCAGTTCTTCAG | miRNA expression |
| miR146a-3p_M_F | TCAGGCACCTgTGAAATTCAGTTCTTCAG | miRNA expression |
| miR146a-3p_SL | gtcgtatccagtgcagggtccgaggtattcgcactggatacgacCTGAAG | miRNA cDNA synthesis |
| miR593-5p_F | CAGCCAGGCATTGCTCAGC | miRNA expression |
| miR593-5p_M_F | ACtAGCCAGGCATTGCTCAGC | miRNA expression |
| miR593-5p_SL | gtcgtatccagtgcagggtccgaggtattcgcactggatacgacGCTGAG | miRNA cDNA synthesis |
| miR1304-3p_F | ATCTCACTGTAGCCTCGAACCCC | miRNA expression |
| miR1304-3p_M_F | GCATCTCACTGTAGCaTCGAACCCC | miRNA expression |
| miR1304-3p_SL | gtcgtatccagtgcagggtccgaggtattcgcactggatacgacGGGGTT | miRNA cDNA synthesis |
| miR548l_F | TCAGGCAAAAAGTATTTGCGGGTTTTGTC | miRNA expression |
| miR548l_M_F | AGCAGGCAAAAAGTATTTGtGGGTTTTGTC | miRNA expression |
| miR548l_SL | gtcgtatccagtgcagggtccgaggtattcgcactggatacgacGACAAA | miRNA cDNA synthesis |
| miR196a-3p_F | ACGGCAACAAGAAACTGCCTGAG | miRNA expression |
| miR196a-3p_M_F | GCACGGCAACAAGAAACTGtCTGAG | miRNA expression |
| miR196a-3p_SL | gtcgtatccagtgcagggtccgaggtattcgcactggatacgacCTCAGG | miRNA cDNA synthesis |
| miR196a-3p_M_SL | gtcgtatccagtgcagggtccgaggtattcgcactggatacgacCTCAGa | miRNA cDNA synthesis |
| miR499a-3p_F | GCAAACATCACAGCAAGTCTGTGCT | miRNA expression |
| miR499a-3p_M_F | CAAACgTCACAGCAAGTCTGTGCT | miRNA expression |
| miR499a-3p_SL | gtcgtatccagtgcagggtccgaggtattcgcactggatacgacAGCACA | miRNA cDNA synthesis |
| miR499b-5p_F | AGGCAACAGACTTGCTGTGATGTTCA | miRNA expression |
| miR499b-5p_M_F | GCAACAGACTTGCTGTGAcGTTCA | miRNA expression |
| miR499b-5p_SL | gtcgtatccagtgcagggtccgaggtattcgcactggatacgacTGAACA | miRNA cDNA synthesis |
| miR499b-5p_M_SL | gtcgtatccagtgcagggtccgaggtattcgcactggatacgacTGAACg | miRNA cDNA synthesis |
| Uni_RT-qPCR_R | CCAGTGCAGGGTCCGAGG | miRNA expression |
| Seq_miR593-5p_F | AGCCTTCACATGAGCCCTG | Sanger sequencing |
| Seq_miR593_5p_R | CCATGGAAACTGGCTATCACT | Sanger sequencing |
| Seq_miR1304-3p_F | ACCTCAACCACTATTTACAAGGT | Sanger sequencing |
| Seq_miR1304-3p_R | AGTAGGAAGTTTCTGCAGTTGG | Sanger sequencing |
| Seq_miR548l_F | AGGCCAGGAAGTATTAATAAGGG | Sanger sequencing |
| Seq_miR548l_R | TGGGATGTAGACTGTGGAGG | Sanger sequencing |
| Seq_miR196a-3p_F | ACCCAGCAACCCAAAGTCTA | Sanger sequencing |
| Seq_miR196a-3p_R | CACTCACAGCTTGTCCTCCT | Sanger sequencing |
| Seq_miR499ab5p_3p_F | TGGGGTGAAAGAGAAGCGTA | Sanger sequencing |
| Seq_miR499ab5p_3p_R | CCATCACCACCACCAAAGTC | Sanger sequencing |
| Seq_miR575_F | TGTGTCTAGGGAGCATGTCT | Sanger sequencing |
| Seq_miR575_R | TCCTGGCAGATGTGGGAAAT | Sanger sequencing |
| Seq_miR449b-5p_F | AGCAGAAAGTACACATAAACACA | Sanger sequencing |
| Seq_miR449b-5p_R | AGAGTTCTGCCACTATGTTTCA | Sanger sequencing |
| Seq_miR146a-3p_F | ATGAGTGCCAGGACTAGACC | Sanger sequencing |
| Seq_miR146a-3p_R | CCTGAGACTCTGCCTTCTGT | Sanger sequencing |

**Supplementary Table 2.** Variants in mature miRNAs found in patients with RASopathies.

| **microRNA acronym** | **SNP ID** | **Minimum Allele Frequency (MAF)** | **Variant Position (chromosome:position)** | **Nucleotide change** | **Zigosity in Patients (P1-P5)** |
| --- | --- | --- | --- | --- | --- |
| hsa-miR-3675-5p | rs202014433 | T=0.2220 (ExAC) | 1:16859005 | C>T | Heterozygous4 |
| hsa-miR-1231 | rs116338160 | A=0.010 (1000G); A=0.00385 (ExAC); A=0.00307 (GnomAD_exome); A=0.0111 (GnomAD) | 1:201808623 | G>A | Heterozygous1 |
| hsa-miR-1231 | rs112032363 | A=0.010 (1000G); A=0.0111 (GnomAD) | 1:201808624 | C>A | Heterozygous1 |
| hsa-miR-6810-5p | rs62182086 | G=0.070 (1000G); G=0.1806 (ExAC); G=0.10951 (GnomAD_exome); G=0.1194 (GnomAD) | 2:218341922 | A>G | Heterozygous4 |
| hsa-miR-6811-3p | rs2292879 | G=0.375 ( 1000G);G=0.3119 (GnomAD) | 2:237510968 | A>G | Heterozygous4,5 |
| hsa-miR-570-5p | rs9860655 | C=0.073 (1000G); C=0.09768 (GnomAD_exome); C=0.1407 (ExAC) | 3:195699434 | T>C | Heterozygous5 |
| hsa-miR-1255b-5p | rs6841938 | A=0.088 (1000G); A=0.1054 (GnomAD); A=0.086 (GnomAD_exome); A=0.103 (ExAC) | 4:36426426 | G>A | Heterozygous4 |
| hsa-miR-1269a | rs73239138 | A=0.394 (1000G); A=0.3533 (GnomAD); A=0.26535 (GnomAD_exome); A=0.28692 (33202/115718, ExAC) | 4:66276902 | G>A | Heterozygous3,5 |
| hsa-miR-575 | rs149186367 | A=0.003 (1000G); A=0.0016 (GnomAD) | 4:82753367 | G>A | Heterozygous4 |
| hsa-miR-449b-5p | rs10061133 | G=0.122 (1000G); G=0.11106 (ExAC); G=0.10695 (GnomAD_exome); G=0.0940 (GnomAD) | 5:55170716 | A>G | Heterozygous1 |
| hsa-miR-146a-3p | rs2910164 | C=0.2946 (GnomAD); C=0.28693 (GnomAD_exome); C=0.27925 (ExAC); | 5:160485411 | C>G | Homozygous1,2,3,4,5 |
| hsa-miR-590-3p | rs6971711 | T=0.0262(1000Genomes); T=0.0057 (GnomAD_exomes); T=0.0223 (GnomAD); T=0.0067 (ExAC) | 7:74191269 | C>T | Heterozygous1 |
| hsa-miR-6875-3p | rs112975788 | G=0.003 (1000G); G=0.00091 (GnomAD_exome); G=0.0032 (GnomAD); G=0.00120 (ExAC) | 7:100868092 | C>G | Heterozygous2 |
| hsa-miR-593-5p | rs73721294 | T=0.027 (1000G); T=0.00500 (GnomAD_exome); T=0.0184 (GnomAD) | 7:128081882 | C>T | Heterozygous1 |
| hsa-miR-6841-3p | rs76347846 | G=0.066 (1000G); G=0.06578 (GnomAD_exome); G=0.0736 (GnomAD); G=0.0945 (ExAC) | 8:24953808 | A>G | Heterozygous2 |
| hsa-miR-7112-5p | rs7012034 | C=0.0078 (GnomAD_exome) | 8:144262731 | T>C | Heterozygous3 |
| hsa-miR-938 | rs12416605 | T=0.125 (1000G); T=0.22244 (GnomAD_exome); T=0.2132 (GnomAD); T=0.22595 (ExAC) | 10:29602331 | C>T | Heterozygous3,5 |
| hsa-miR-608 | rs4919510 | G=0.364 (1000G); G=0.26583 (GnomAD_exome); G=0.2718 (GnomAD); G=0.27083 (ExAC) | 10:100975021 | C>G | Homozygous2 |
| hsa-miR-1304-3p | rs2155248 | *G=0.121(1000G); G=0.03986 (ExAC) | 11:93733700 | G>T | Homozygous2,3,4,5 |
| hsa-miR-548l | rs13447640 | A=0.052 (1000G); A=0.0431 (GnomAD) | 11:94466555 | G>A | Heterozygous5 |
| hsa-miR-196a-3p | rs11614913 | T=0.333 (1000G); T=0.39251 (GnomAD_exome); T=0.3532 (GnomAD); T=0.42204 (ExAC) | 12:53991815 | C>T | Heterozygous3 |
| hsa-miR-7107-3p | rs3817551 | G=0.335 (1000G); G=0.39231 (GnomAD_exome); G=0.3636(GnomAD); G=0.38548 (ExAC) | 12:121444295 | T>G | Heterozygous2,4 |
| hsa-miR-7107-5p | rs55671311 | G=0.032(1000G); G=0.06088(GnomAD_exome); G=0.0497(GnomAD); G=0.06294 (ExAC) | 12:121444338 | C>G | Heterozygous2 |
| hsa-miR-6763-3p | rs3751304 | *C=0.267(1000G); C=0.27006 (GnomAD_exome); C=0.3019 (GnomAD); C=0.2807 (ExAC) | 12:132582046 | C>T | Homozygous3 |
| hsa-miR-92a-1-5p | rs9589207 | A=0.024(1000G); A=0.0207 (GnomAD); A=0.00663(ExAC) | 13:91351335 | G>A | Heterozygous3 |
| hsa-miR-412-3p | rs61992671 | G=0.189(1000G); G=0.35255(GnomAD_exome); G=0.3479 (GnomAD); G=0.34804(ExAC) | 14:101065517 | A>G | Heterozygous1,3,5 |
| hsa-miR-627-5p | rs2620381 | C=0.084 (1000G); C=0.0439(GnomAD_exome); C=0.0426 (GnomAD);C=0.0479  (ExAC) | 15:42199650 | A>C | Heterozygous3 |
| hsa-miR-6863 | rs12708966 | A=0.054 (1000G); A=0.03162 (GnomAD_exome); A=0.0341 (GnomAD); A=0.03105 (ExAC) | 16:56904332 | G>A | Heterozygous4 |
| hsa-miR-6778-3p | rs377036954 | C=0.00013 (GnomAD_exome); C=0.0007(GnomAD); C=0.0002 (ExAC) | 17:18340819 | G>C | Heterozygous2 |
| hsa-miR-6868-3p | rs7208391 | C=0.466(1000G);G=0.4892 (GnomAD); | 17:76098024 | C>G | Heterozygous1,3,4 |
| hsa-miR-4743-5p | rs141766192 | T=0.004 (1000G); T=0.00078 (GnomAD_exome); T=0.0029 (GnomAD); T=0.00091 (ExAC) | 18:48670606 | C>T | Heterozygous3 |
| hsa-miR-4745-5p | rs10422347 | T=0.090 (1000G); T=0.09857 (GnomAD_exome); T=0.0772 (GnomAD); T=0.09423 (ExAC) | 19:804959 | C>T | Heterozygous2,3,4 |
| hsa-miR-6885-5p | rs78293125 | G=0.027 (1000G); G=0.05874 (GnomAD_exome) ; G=0.0572 (GnomAD) ; G=0.06317 (ExAC) | 19:6389688 | A>G | Heterozygous4 |
| hsa-miR-6887-5p | rs1688017 | A=0.343 (1000G); A=0.35639 (GnomAD_exome); A=0.3606 (GnomAD); A=0.36441(ExAC) | 19:35122719 | G>A | Heterozygous2,5 |
| hsa-miR-6796-3p | rs3745198 | G=0.383 (1000G); G=0.45223 (GnomAD_exome); G=0.3435 (GnomAD) | 19:40369893 | C>G | Heterozygous3,4,5 |
| hsa-miR-6796-3p | rs3745199 | G=0.383 (1000G) | 19:40369903 | C>G | Heterozygous3,4,5 |
| hsa-miR-6801-3p | rs10412196 | C=0.153 (1000G); C=0.10905 (GnomAD_exome); C=0.1606 (GnomAD); C=0.1952 (ExAC) | 19:52222085 | T>C | Heterozygous3,5 |
| hsa-miR-499a-3p | rs3746444 | G=0.184 (1000G); G=0.19593 (GnomAD_exome); G=0.1817 (GnomAD); G=0.20133 (ExAC) | 20:34990448 | A>G | Heterozygous1,3,5 |
| hsa-miR-499b-5p | rs3746444 | G=0.184 (1000G); G=0.19593 (GnomAD_exome); G=0.1817 (GnomAD); G=0.20133 (ExAC) | 20:34990448 | A>G | Heterozygous1,3,5 |
| hsa-miR-646 | rs6513497 | G=0.191(1000G);G=0.1735 (GnomAD); G=0.11990 (ExAC) | 20:60308547 | T>G | Heterozygous4,5 |
| hsa-miR-503-3p | rs373252206 | T=0.000 (1000G); T=0.00029 (GnomAD_exome); T=0.0013 (GnomAD); T=0.0005 (ExAC) | X:134546351 | C>T | Heterozygous3 |

**Supplementary Table 3.** Target genes of selected miRNAs

| **Supplementary Table 2** | **gene_symbol** |
| --- | --- |
| hsa-miR-1304-3p | A2ML1*, ABCA9, ABCB5, ABHD12B, ABL2, ABRAXAS1, ACOT2, ACPP, ACSL6, ADAMTS4, ADH6, ADM2, AGO3, AICDA, AIDA, AKAP10, AKR1D1, AKR7A2, ALDH1A3, ALDH6A1, ALDOA, ALG1, ALKAL1, ALOX5AP, ALX1, AMMECR1L, ANAPC16, ANKRD26, ANKRD9, ANKS4B, AP1G1, AP1S1, AP3M2, APCDD1, APOL1, APOPT1, APTX, AQR, AREL1, ARHGAP29, ARID1A, ARL10, ARMC1, ARNT, ARSK, ASB3, ATAD3C, ATF7IP, ATG14, ATG5, ATOH8, ATXN1L, ATXN7, BCL2L2, BCL7B, BDP1, BHLHE40, BHMT2, BMF, BMP7, BMPR1A, BMPR2, BOD1, BORCS7, BPNT1, BPTF, BRD2, BRMS1L, BSDC1, BTBD19, BTN3A2, C11orf54, C11orf70, C12orf43, C17orf105, C17orf75, C19orf73, C1orf158, C2orf91, C3, C5orf64, C6orf132, C7orf55-LUC7L2, C7orf65, C8orf17, CABP4, CACNG8, CADM1, CAPZA2, CARS, CAVIN4, CBLN3, CCDC36, CCDC68, CCDC69, CCL22, CCNG2, CCR9, CD109, CD1D, CD69, CDC14B, CDC42SE2, CDH8, CDK9, CDKN3, CDNF, CEACAM8, CENPK, CENPM, CEP57L1, CEP89, CFHR5, CHMP1B, CHRNB1, CIAO1, CISD2, CLDN1, CLGN, CLK4, CNBP, CNDP1, CNKSR3, CNN3, CNOT6L, CNST, COL13A1, COL25A1, COMMD5, COMMD9, COQ7, COX20, CRK, CTCFL, CXCR2, CXCR5, CXorf56, CYCS, CYP27C1, DBF4, DBN1, DBT, DCAF7, DCP1A, DDB1, DDX19B, DENND4C, DENND5B, DFFA, DGKE, DGKH, DHTKD1, DIRAS1, DIS3L, DMP1, DMRT2, DNAJB13, DNAJC22, DNAJC28, DNM3, DPPA3, DRAXIN, DSG3, DSTYK, DYRK1A, EDARADD, EDC3, EDEM3, EEF2K, ELOVL4, ELP2, EMCN, ENSA, ERAP2, ERGIC1, ERVMER34-1, EXO5, EXOSC10, F2RL1, FADS6, FAM120A, FAM120AOS, FAM124A, FAM155B, FAM168A, FAM177A1, FAM19A1, FAM20B, FAM212B, FAM229B, FAM71F2, FAM83D, FAM96A, FAR2, FBXL16, FBXO41, FBXO47, FBXW8, FFAR2, FGFR1, FGFR1OP2, FITM2, FLT3LG, FNDC5, FOXI1, FOXK1, FPR1, FUT11, FXN, FZD3, G3BP1, G6PC, GAS6, GATA6, GATAD1, GBP7, GDE1, GDF11, GEMIN6, GFPT1, GINM1, GJA9, GJD4, GK5, GLO1, GLYAT, GMCL1, GMEB1, GNB4, GNPNAT1, GP2, GPR132, GPR155, GPR158, GPR75-ASB3, GPR82, GPR88, GRIN2B, GRK3, GRPEL2, GRSF1, GSR, GSTM3, GTF3C4, GTF3C6, GTPBP10, H6PD, HAUS3, HDC, HEATR5A, HES2, HEYL, HINFP, HIPK1, HIST1H2AH, HLTF, HM13, HMGB1, HNMT, HNRNPUL1, HOOK3, HOXA9, HOXB5, HPSE, HSD17B12, HTR2C, HUS1, HYPK, IBA57, ICA1L, IDE, IDS, IFNAR1, IGSF5, IGSF6, IKZF3, IL10RB, IL1RL1, IL6R, ILDR1, INO80, INPP4A, IRAK1, IRAK4, IRX3, ISCA2, ISY1, ITGB1BP1, ITPRIPL1, KANK2, KAT5, KBTBD2, KBTBD6, KCMF1, KCNJ6, KCTD18, KDM2B, KDM5A, KIAA0232, KIAA0586, KIAA1143, KIAA1191, KIAA1468, KIAA1586, KIAA1614, KIRREL2, KLF5, KLHL15, KLHL7, KLK11, KLK7, KPNA2, KRBA2, LAX1, LCAT, LDHA, LDLR, LHPP, LIAS, LILRA2, LIMS1, LINC00598, LINC00632, LMAN2L, LMCD1, LMLN, LNPK, LONP2, LRAT, LRCH3, LRIF1, LRIG2, LRP10, LRRC2, LRRC3C, LRRC8B, LRRD1, LRRN3, LSM10, LUC7L2, LYN, LYPD5, LYRM4, LYRM7, MAP3K20, MAP3K9, MAP7, MAVS, MCM4, MCM8, MCOLN2, MCTS1, MED7, MELK, MESD, MFSD14C, MICA, MIEF1, MLXIP, MMGT1, MOG, MOGAT3, MPDU1, MPZ, MRNIP, MRPL17, MRPL36, MSANTD4, MSRB1, MSRB3, MTA3, MTERF1, MTMR10, MYBL1, MYLK3, MYO1F, MYO5A, MYO5B, MYOZ2, MYOZ3, NAALADL1, NACC1, NAGK, NAP1L6, NCKAP1, NDOR1, NELFCD, NEURL1B, NFATC2, NHS, NIPAL1, NKAP, NKD1, NLK, NOL10, NOM1, NOTCH2, NPTXR, NQO2, NR3C1, NRIP1, NRN1, NSD2, NUP93, NWD1, NXPE2, NXPE3, NYAP2, OGFOD1, OLA1, OR1C1, ORAI2, ORC6, OTUB2, OXA1L, PACS1, PACSIN2, PANK2, PANK3, PARD3B, PARVB, PCDH17, PCDHA6, PCNX2, PCSK9, PDE3A, PDE4C, PDE6A, PDLIM3, PDP2, PEX26, PEX5L, PFAS, PHAX, PHF12, PHLDA3, PI4K2B, PIAS2, PIGG, PIGP, PIGX, PIKFYVE, PIWIL1, PLA2G12A, PLA2G16, PLCXD3, PLEKHM3, PLEKHO1, PLEKHS1, PLIN5, PLN, PLPBP, PLXDC2, PLXNA2, PNPLA3, PNPT1, PNRC1, POC1A, POLR2D, POM121L7P, POTED, POU2F2, POU6F1, PPEF1, PPIC, PPL, PPM1K, PPP1R10, PPP4R2, PPP5D1, PPRC1, PRAME, PRAMEF1, PRDM10, PRICKLE2, PRKAR2A, PRKCI, PROSER2, PRPF38A, PRPF4, PRR11, PRR23A, PRRT3, PSMA8, PSMB2, PSMB9, PSPH, PTCD3, PTCHD3, PTDSS1, PTGFRN, PTGS1, PTPN14, PURB, PVR, QRSL1, RAB10, RAB11FIP1, RAB27A, RAB38, RAB40B, RABAC1, RACGAP1, RAD50, RAD51L3-RFFL, RBM48, RBM4B, REEP5, REL, RFFL, RFT1, RGS17, RHBDL2, RHOA, RHOU, RIOK2, RMND1, RNASE4, RNF11, RNF123, RNF14, RNF19B, RNF222, ROMO1, RPL10A, RPL34, RREB1, RRP36, RSBN1L, RSPH3, RTTN, SAMD5, SAV1, SCAF11, SCO1, SCP2, SCRG1, SCYL2, SEC14L5, SEC23B, SEC61A2, SEC62, SEMA3E, SEMA5A, SERF2, SERPING1, SERTM1, SF3B1, SGO1, SGPP2, SH3BGRL, SHE, SHISA9, SIGLEC9, SIK2, SIRPG, SKAP2, SLC10A6, SLC16A10, SLC19A3, SLC1A5, SLC25A16, SLC25A19, SLC25A33, SLC25A45, SLC25A51, SLC35B4, SLC35E1, SLC35F5, SLC37A2, SLC38A9, SLC43A1, SLC43A2, SLC7A2SNAP29, SMAGP, SMG1, SMIM19, SMYD4, SNRPD1, SNTB2, SNW1, SOWAHA, SOWAHB, SOX7, SP110, SPATA21, SPATA5, SRARP, SRF, SRSF2, SRSF7, ST3GAL1, ST3GAL2, STAC, STARD13, STARD3NL, STC1, STK17B, STRN3, STT3B, STX6, STXBP2, SUCO, SUSD1, SUSD4, SVOP, SYAP1, SYNPO2L, SYNRG, TAF1B, TARS2, TATDN2, TBATA, TCEANC2, TECPR1, TEKT4, TESK2, TEX22, TGFB2, THEM4, THRB, THUMPD2, TIGAR, TIMELESS, TIMM10, TIMM17A, TIMM29, TIMM8A, TIRAP, TMCO1, TMEM132B, TMEM168, TMEM19, TMEM216, TMEM243, TMEM250, TMEM260, TMEM67, TMEM72, TMEM8B, TMIGD2, TNFAIP8L1, TNFAIP8L3, TNFRSF13B, TNRC6B, TPCN2, TPGS1, TPRG1L, TRAPPC13, TRIB1, TRIM10, TRIM4, TRIM65, TRIM66, TRIM72, TRMT10B, TRPM7, TRUB2, TSPAN14, TSPAN31, TSPAN6, TTC17, TTC31, TTC4, TTLL12, TTPAL, TTR, TUBD1, TXLNA, TXNL1, TXNRD3NB, TYR, UBE2G2, UBTD2, UBXN2A, UEVLD, UGGT1, UGT2B4, UNC5D, UROS, USHBP1, USP13, USP14, USP50, VEZT, VHL, VPS33B, VPS37A, VSIG1, VSIG2, VSTM4, VWC2L, WASF2, WDFY3, WDR12, WDR78, WDR92, WIPF3, WWC1, XPO5, XRCC2, YEATS2, YIPF4, YIPF5, YY1, ZBED1, ZBTB24, ZBTB25, ZC2HC1C, ZC3H4, ZDHHC22, ZDHHC8, ZER1, ZFP14, ZFP82, ZFP91, ZKSCAN1, ZMAT4, ZMYM3, ZNF101, ZNF106, ZNF135, ZNF177, ZNF207, ZNF311, ZNF320, ZNF333, ZNF347, ZNF384, ZNF417, ZNF419, ZNF426, ZNF430, ZNF431, ZNF439, ZNF485, ZNF487, ZNF513, ZNF529, ZNF546, ZNF551, ZNF554, ZNF556, ZNF557, ZNF559-ZNF177, ZNF561, ZNF566, ZNF652, ZNF655, ZNF665, ZNF682, ZNF708, ZNF780A, ZNF799, ZNF8, ZNF829, ZNF841, ZNF85, ZWINT, ZZZ3 |
| hsa-miR-146a-3p | A1CF, ABCA9, ABCB1, ABCB10, ABL2, ACKR3, ACOX1, ACTL10, ACTN4, ADA2, ADAMDEC1, ADAMTS1, ADAMTS17, ADAMTS5, ADARB1, ADCY2, ADNP2, ADPRH, AFAP1, AFF4, AGBL3, AHCYL1, AHCYL2, AIFM1, AKIRIN1, ALAS1, ALCAM, ALG10, ALOX12-AS1, ALPK1, ANAPC16, ANKRD1, ANKRD40, ANKRD9, APEX1, APIP, AREL1, ARHGEF12, ARID4A, ARL10, ARL8B, ARRDC2, ASB7, ATE1, ATF2, ATG13, ATG2B, ATG7, ATL3, ATP6V1C2, ATP8A1, ATXN1, ATXN1L, B9D1, BAG3, BAZ2A, BCAT1, BCCIP, BEAN1, BMP2, BNC2, BNIP3L, BOD1L2, BRD3, BTF3L4, BUD13, C11orf44, C11orf53, C11orf95, C14orf28, C17orf75, C1orf50, C22orf23, C2orf50, C2orf69, C3orf67, C5orf63, C8orf46, C8orf86, C9orf64, CALML4, CASC4, CASP7, CBR1, CCDC39, CCDC73, CCL1, CCM2, CCNT2, CD207, CD2AP, CD300E, CD44, CDC27, CDCA7, CDH12, CDH8, CDKN2AIP, CEMIP, CEP57, CEP85, CFAP47, CFL2, CHD2, CHD9, CHM, CIPC, CLDN16, CLEC6A, CLGN, CLLU1, CLVS2, CLYBL, CNTD1, COMMD8, CORO1C, CP, CPEB4, CPLX2, CREBRF, CRYZ, CSNK1G1, CUTC, CUX2, CXCL9, CXCR4, CYB5B, CYB5R2, DAB2, DARS2, DDHD1, DDX3X, DEPTOR, DERL2, DIRC2, DIXDC1, DMRT3, DNAJC19, DNAJC8, DNM3, DNMT3A, DPAGT1, DST, DUSP13, DUSP4, DZIP3, EBF3, ECHDC1, EED, EEF2, EGFL8, EGR2, EIF4E3, ELAVL1, ELMSAN1, ELN, ELOC, ELSPBP1, EMC8, EPB41L3, EPS15, ERBB2, ERC1, ERCC6L2, ETNK1, ETS1, EVA1A, EXOC5, EYA4, F11R, FAM107B, FAM126A, FAM126B, FAM169A, FAM20A, FANCL, FBXO32, FCGR3A, FCGR3B, FCRL1, FCRL5, FDFT1, FEM1B, FGD2, FMR1NB, FOXC1, FOXD4L5, FOXE1, FOXN2, FRY, FSIP1, FUCA1, FZD4, G0S2, GADL1, GALT, GAST, GBP6, GCC2, GEN1, GFPT2, GMEB2, GNAQ, GNB5, GOLGA4, GON7, GPATCH2L, GPR107, GPR12, GREB1, GRIA3, GSN, GTDC1, GTF2A1, GYPE, H2BFM, HABP2, HCAR2, HELLS, HEY2, HHEX, HIBADH, HIRIP3, HIST1H1B, HIST1H1C, HIST1H1D, HIST1H1E, HIST1H2AA, HIST1H2AD, HIST1H2AE, HIST1H2AH, HIST1H2AI, HIST1H2AJ, HIST1H2BA, HIST1H2BB, HIST1H2BE, HIST1H2BG, HIST1H2BH, HIST1H2BL, HIST1H2BO, HIST1H3A, HIST1H3B, HIST1H3C, HIST1H3D, HIST1H3F, HIST1H3H, HIST1H3J, HIST2H2AA3, HIST2H2AA4, HIST2H2BE, HIST2H2BF, HIST2H3A, HIST2H3C, HIST2H3D, HIST3H2BB, HLA-DMB, HLA-DOA, HLCS, HLF, HMGCR, HNRNPA0, HNRNPK, HOXC4, HOXD3, HPSE, HSF2, HSPD1, ICOSLG, ID2, IDI2, IDNK, IFI44L, IGFBP3, IGFL3, IGHMBP2, IGSF3, IKZF1, IL5, IL6, ILF3, IRS2, IRX4, ITGB7, ITGB8, ITPKC, KALRN, KANSL1, KANSL3, KCNC2, KCTD2, KDELR3, KDM5A, KHNYN, KIAA1324, KIDINS220, KLF12, KLHL14, KLHL21, KPNA1, KPNA4, KPNA6, KRIT1, KRT12, KTN1, L3HYPDH, LACTB2, LAP3, LARP4, LCOR, LDAH, LGI2, LHFPL5, LILRB2, LIN28B, LIPT2, LITAF, LMNB2, LMO3, LMO4, LOC101928882, LRIG1, LRRC49, LTBR, LUZP4, LYRM2, MAFG, MANEA, MAP3K2, MAP3K20, MAPRE1, MARCH6, MBLAC2, MC2R, MCOLN2, MDGA2, MDM4, MED10, MED14, MEF2C, MEX3A, MICU3, MINDY1, MINDY2, MLF1, MME, MNAT1, MNT, MOBP, MPHOSPH8, MPP7, MPZL2, MRPL11, MRPL35, MRVI1, MST1R, MSTN, MTERF1, MTMR4, MXD1, MYL12A, NAP1L2, NAXE, NCAM1, NCOA2, NCOA3, NDNF, NEK2, NEK5, NEK7, NEU1, NEUROD4, NFAT5, NFU1, NFYB, NHLH2, NHSL1, NIFK, NLRP9, NNT, NOL8, NOVA1, NPHS2, NRCAM, NRK, NSUN7, NTRK2, NUFIP2, NUS1, NXNL2, NYX, ONECUT2, OPCML, OPHN1, OPRK1, OR7A17, OSBPL3, OSGIN2, OTULIN, OXA1L, P2RX7, PACRGL, PACSIN2, PAGR1, PARD3B, PARPBP, PAXBP1, PCDH17, PCDH19, PCDHB10, PCGF5, PCM1, PCSK2, PCSK5, PDE4D, PDE4DIP, PDIA3, PDS5A, PEX11B, PHB, PKD2L1, PKIA, PLAGL2, PLCXD3, PLD5, PLEKHA7, PLEKHH2, PLEKHS1, PLP2, PLPBP, PLPP6, PLXNA1, PMEPA1, PNPLA1, POGLUT1, POLR1D, POU2F3, PPP1R2, PPP1R3B, PPP1R9A, PPP2R5C, PRCP, PRICKLE2, PRKAB2, PRKG2, PRLR, PROX1, PRR20A, PRR20B, PRR20C, PRR20D, PRR20E, PRR9, PRRT4, PSD4, PSG4, PTCH1, PTPDC1, PTPN12, PTPN14, PTTG1, RAB22A, RAB34, RAB3B, RAB8A, RAF1*, RAG1, RANBP17, RAP1GAP, RAPGEF2, RASL12, RASSF8, RB1, RBFOX2, RBM17, RBM24, RC3H1, RECK, RECQL, RHCE, RICTOR, RIOK3, RIOX1, RND3, RNF125, RNF19B, RNF44, RPAP1, RREB1, RRS1, RUNX2, RWDD4, SARM1, SAT2, SCN9A, SCOC, SELE, SELENOT, SEMA3E, SEMA5A, SEPT6, SEPT8, SERBP1, SERINC1, SERTAD2, SERTAD4, SESN3, SET, SETX, SFMBT2, SFTPA2, SGCG, SGPL1, SH3BGRL2, SH3BP5, SH3PXD2A, SH3TC2, SHISA6, SIDT2, SIX1, SKIL, SLC16A6, SLC24A4, SLC31A1, SLC39A8, SLC44A1, SLC4A10, SLC8A1, SLC9A3R2, SLC9A7, SLFN5, SMC1B, SNAI2, SNAP23, SNAP91, SNX13, SNX27, SOD1, SOD2, SORT1, SOX17, SPAG11A, SPATS2, SPCS1, SPECC1L, SPEN, SPIN1, SPINK7, SPOCK1, SPPL3, SPRED1*, SPRYD7, SPTLC2, SRGAP3, SRP9, SRPK1, SRRM1, SSFA2, STRADA, STX5, STXBP6, SUCO, SUMO1, SYNCRIP, TAF11, TAF4, TANC2, TAOK1, TAOK2, TBC1D4, TCF12, TCF4, TCF7, TEAD4, TEP1, TFE3, TGFA, TIA1, TLX1, TM7SF2, TMA16, TMCC3, TMEFF1, TMEM167A, TMEM199, TMEM268, TNS2, TNS3, TPP2, TRABD, TRAF3IP1, TRAK1, TRAPPC11, TRAPPC8, TRIAP1, TRIB3, TRIM34, TRIM36, TRIM39, TRIM59, TRIM6-TRIM34, TRIM66, TRIM69, TRMT2B, TRPM4, TTC14, TTC39B, TTLL6, TXNIP, TXNL1, UBALD1, UBAP1, UBASH3B, UBE2H, UBE2U, UBFD1, UBN2, UGT2B11, UGT2B28, UGT2B7, ULK2, UNC13A, USH2A, USP18, USP37, USP42, UTP15, VANGL1, VAPB, VAV3, VCX2, VCY, VCY1B, VMAC, VNN2, VPS26B, VWA1, WARS2, WBP1L, WDR33, WDR7, WDR73, WDR78, WT1, WTAP, WWTR1, XIRP1, YAF2, YBX2, YIPF2, YME1L1, YTHDF2, ZBED6CL, ZC2HC1C, ZC3H15, ZC3H4, ZC3HAV1, ZEB2, ZFP1, ZFP69B, ZFX, ZFYVE26, ZKSCAN1, ZMYM2, ZNF107, ZNF140, ZNF148, ZNF175, ZNF180, ZNF185, ZNF225, ZNF23, ZNF24, ZNF250, ZNF264, ZNF286A, ZNF302, ZNF322, ZNF330, ZNF35, ZNF384, ZNF391, ZNF395, ZNF415, ZNF428, ZNF441, ZNF487, ZNF510, ZNF544, ZNF547, ZNF566, ZNF607, ZNF611, ZNF616, ZNF667, ZNF675, ZNF678, ZNF704, ZNF708, ZNF709, ZNF77, ZZEF1 |
| hsa-miR-196a-3p | AAED1, AATK, ABCD1, ABCF3, ABI2, ACOT11, ACTR1A, ADAMTS17, ADD1, ADGRD1, ADORA2B, AEN, AHCYL2, AK5, AKR7A2, ALKBH5, AMIGO2, AMMECR1L, ANKIB1, ANKRD13A, ANKRD20A2, ANKRD20A4, AP1M1, AP3M1, APLF, ARF4, ARID1A, ARMCX6, ARRDC3, ARSE, ASB15, ASPHD2, ASRGL1, ATF7, ATG12, ATL2, ATP1B3, ATP2B4, ATP8A2, ATRAID, AZIN1, B4GALT5, B4GALT7, BAG3, BCAS4, BCL7C, BEX3, BHLHE41, BRAF*, BROX, BRWD3, C12orf73, C17orf105, C18orf32, C1GALT1, C1orf198, C1orf35, C1orf50, C2orf88, C8orf37, C9orf47, C9orf72, CA4, CACNA2D1, CACNB2, CAD, CAMK4, CAPZB, CARD6, CARF, CASP16P, CCDC144NL, CCDC28A, CCDC8, CCR8, CD300A, CDC25B, CDC37L1, CDC42BPA, CDC42EP1, CDCP1, CDK2, CDKN2AIP, CENPH, CENPL, CENPO, CEP104, CEP152, CFAP97, CGGBP1, CHAF1B, CHIA, CHMP5, CHST1, CIB1, CKS1B, CLPTM1L, CNBP, CNOT6, CNTFR, COA4, CORO1C, COX6B1, CRCP, CRIP1, CRNKL1, CTSG, CUL4A, CXCL10, CYB5B, CYP1A2, CYP27C1, CYP4F3, CYYR1, DAB2, DCAF12L1, DCTN4, DDAH1, DDX19B, DDX52, DENND1B, DFFB, DHFR, DLG2, DNAH10OS, DNAJC28, DOCK7, DPY19L3, DSEL, DUSP22, DUSP4, DYNC1I1, EFCAB1, EFNA4, EFNB1, EGLN1, EGR3, EIF1AD, EIF3A, EIF3C, EIF3CL, EIF3H, EIF3J, EIF4G2, EIF4H, EIF5A, EIF5A2, EIF5AL1, ELAVL4, ELOC, ELOVL6, EMC1, ENO4, EPAS1, EPB41, ERCC1, ERMP1, EXOSC2, EYA3, F2RL2, FAM114A2, FAM117B, FAM122B, FAM133B, FAM136A, FAM155B, FAM210A, FAM50B, FAM83F, FBXL3, FBXO45, FBXO47, FGF2, FGF9, FIGNL1, FLRT3, FOXF2, FOXL2NB, FOXN2, FOXO3, FRS2, FTHL17, FUT1, GAB1, GADL1, GAGE1, GAPVD1, GART, GFOD2, GK5, GLTP, GMNC, GMPR, GOLPH3, GON7, GPCPD1, GPR12, GPR162, GPR75, GPR85, GRM5, GTF2A2, GTF2B, GUCD1, H6PD, HACD2, HACE1, HAUS3, HBP1, HEATR1, HECTD1, HERPUD1, HNMT, HNRNPLL, HRH4, HS6ST2, HSBP1, HSPA12A, ID3, IDS, IGDCC3, IKZF3, IL12RB1, IL32, IL36G, IMPG1, INO80C, INTS13, INTS7, IQCH, IQSEC2, IRAK1BP1, IRGQ, ITGA2, ITPRIPL2, JAZF1, KANK2, KANSL3, KCNE4, KIAA1755, KIF18A, KIF19, KLF10, KLHDC8A, KLHL11, KNSTRN, LAMC3, LDLRAD3, LFNG, LGSN, LIMD1, LINGO1, LMCD1, LPIN3, LRRD1, LSM5, LSP1, LTB4R2, LY6D, LYRM2, MACROD2, MALL, MAP7D2, MAPK1IP1L, MAPRE1, MARCH6, MBNL1, MCM5, ME1, MEIS2, MLH1, MME, MRPS10, MRPS16, MRPS18B, MRPS21, MRRF, MSANTD2, MSRB3, MTCH2, MTF2, MTHFR, MTMR3, MTSS1, MTX3, MXRA8, MYADM, MYL12A, MYLK3, NAPB, NCALD, NCKAP1, NDFIP1, NDP, NEUROD1, NF1*, NHLRC3, NICN1, NPLOC4, NPPA, NQO1, NR3C1, NRP2, NRXN1, NTRK2, NTRK3, NUFIP2, NUP43, OLR1, OR7D2, ORC1, OTULIN, PABPC4L, PAPSS2, PARP2, PAX9, PCBD2, PCGF3, PCGF6, PCIF1, PDK3, PEX2, PHF7, PHPT1, PI4K2B, PITPNA, PLAC8, PLAT, PLAUR, PLIN2, PLK4, PLPP6, PLXNA1, PLXNA3, PMP2, PNKD, POFUT2, PON1, PPEF2, PPFIBP1, PPM1A, PPP3R1, PRAMEF4, PRKCI, PRMT8, PRND, PROSER2, PRPF3, PRR23C, PRRG3, PRSS21, PRSS35, PTP4A1, RAB10, RAB11FIP5, RAB30, RAB32, RABGAP1, RAF1*, RAP1A, RAP2C, RAPGEF1, RAPGEFL1, RBBP9, RD3, REEP5, RETREG3, RHCE, RHNO1, RHPN2, RNF125, RPL13A, RPL7L1, RPRD1A, RPS15A, RPS26, RPUSD4, RSPO2, RTL8C, SAMD4A, SC5D, SCUBE3, SDC1, SEC31A, SERINC1, SGO1, SH3D19, SHE, SIGLEC9, SIX4, SKA1, SLC11A1, SLC11A2, SLC24A4, SLC25A13, SLC25A28, SLC25A38, SLC29A1, SLC30A8, SLC35E2, SLC38A11, SLC45A3, SMAD2, SMARCE1, SMTNL2, SNCA, SNRPD3, SORBS2, SOSTDC1, SOX7, SPIN2A, SPIN2B, SPIRE1, SPRED2*, SPRY1, SPRY4, SPTBN1, SRF, SRM, SRRM1, SSH2, STARD7, STK4, STOM, SUSD5, SYDE1, SYPL1, TATDN3, TBC1D15, TCEAL7, TCTN2, TERF1, TEX2, TFAP2A, TFDP2, TFDP3, THBS2, TIGD6, TIRAP, TMEM128, TMEM159, TMEM178A, TMEM19, TMEM78, TMPRSS11BNL, TNPO1, TPH2, TPM3, TPMT, TRAIP, TRAPPC2, TRAPPC2B, TRIM16L, TRIM27, TRIM4, TRIM62, TRMT61A, TRPC4AP, TRPC5, TRPV1, TSEN2, TSKU, TSPAN1, TSPAN12, TSTD2, TTC39B, TTC39C, TTC4, TUBB2B, TUBB6, TWISTNB, TXLNA, TYMS, TYW3, UBA2, UBE2L3, UBXN2A, UFL1, UGDH, UQCRFS1, USO1, UTP11, VAMP4, VANGL1, VAV3, VEGFC, VHL, VHLL, VMA21, WASL, WBP11, WBP2, WDR12, WDR13, WFS1, WT1, WWTR1, XKR4, XRCC6, YIPF4, ZBTB37, ZBTB44, ZBTB8A, ZBTB8B, ZC3HAV1, ZDHHC24, ZNF10, ZNF177, ZNF234, ZNF286A, ZNF286B, ZNF292, ZNF331, ZNF383, ZNF436, ZNF451, ZNF507, ZNF529, ZNF549, ZNF556, ZNF561-AS1, ZNF574, ZNF652, ZNF704, ZNF749, ZNF844 |
| hsa-miR-449b-5p | AASDHPPT, ABCD1, ABCF3, ABLIM3, ABR, ABRAXAS2, ACBD3, ACKR1, ACSL1, ACSL4, ACSM2B, ACTL10, ACTR1A, ADAM22, ADAMTSL4, ADD2, ADGRG2, ADGRG3, ADGRL1, ADIPOR2, ADK, ADO, ADRA1D, AFF4, AGO4, AGTR1, AGTRAP, AHCYL2, AK3, AK4, ALCAM, ALDOA, ALG6, ALS2CL, AMER1, AMER2, AMOTL2, ANHX, ANK2, ANK3, ANKRD52, ANKS1A, ANP32A, ANP32B, ANXA5, AP1B1, AP1S2, AP2S1, AP3S2, APH1A, APLNR, APOPT1, AQP8, AR, AREG, ARHGAP1, ARHGAP26, ARHGAP36, ARHGDIB, ARHGEF33, ARHGEF9, ARID4A, ARID4B, ARL8B, ARPP19, ARSJ, ASB1, ASB4, ASIC2, ASPHD2, ASXL2, ATG2A, ATG4B, ATG9A, ATMIN, ATP5S, ATP6V0B, ATP6V1C1, ATP6V1E1, ATPAF1, ATRIP, ATXN1L, ATXN7, ATXN7L3, AXL, B3GALNT1, B3GALT5-AS1, B3GAT1, B3GAT3, B4GALT2, BAALC, BAZ2A, BBS1, BCAN, BCL2L13, BCL6, BEND7, BEST1, BEST2, BMP3, BNC2, BRAT1, BRINP1, BRPF3, BTBD11, BTBD18, C11orf54, C12orf54, C12orf57, C14orf28, C16orf58, C18orf32, C1orf116, C1orf159, C1QC, C22orf23, C2CD2, C3orf58, C3orf70, C8orf37, C9orf47, CA7, CACHD1, CACNA1E, CACNB1, CACNB3, CALB1, CALCA, CALCB, CALCOCO2, CALCR, CALHM1, CALN1, CAMSAP1, CAMTA1, CAPN6, CAPN9, CASKIN2, CASP2, CBFA2T3, CBLN4, CBX2, CBX3, CCDC36, CCDC50, CCDC85A, CCL22, CCND1, CCNE2, CD47, CD99L2, CDA, CDC25A, CDC37, CDH9, CDK18, CDK4, CDK6, CDKN1B, CDKN1C, CDRT1, CELF2, CELF6, CENPB, CEP19, CERS6, CFAP43, CFD, CFTR, CHD1, CHM, CHMP7, CHST10, CHST12, CLCN3, CLDN15, CLDN18, CLIC5, CLIP3, CLOCK, CNOT4, CNOT6L, CNTN2, CNTN3, CNTNAP1, CNTNAP2, CNTNAP4, COG3, COL12A1, COL5A2, COMMD9, COPS7B, COPZ1, CORO1C, CPEB3, CPLX2, CPSF6, CR2, CRABP2, CRB3, CREB5, CREBRF, CRHR1, CRIP2, CRTC1, CRY2, CSF1R, CSNK1G3, CTCFL, CTDSP2, CTIF, CTNND1, CTNND2, CTSB, CUEDC1, CXCL16, CXCR3, CYBRD1, CYP4F3, CYREN, CYTH3, DAAM1, DAB2IP, DAGLA, DAPK2, DBNDD1, DBNL, DCAF7, DCTN5, DCX, DDA1, DDX10, DDX17, DDX19B, DEDD2, DENND1A, DGAT1, DGAT2L6, DGKZ, DHRS13, DIXDC1, DLEC1, DLL1, DMAC2, DMRTC1B, DMWD, DNAAF3, DNAJB1, DNAJC16, DNM1L, DNM3, DNMBP, DOCK9, DPF2, DPH6, DPP3, DPYSL4, DRC7, DRD2, DSC3, DYNC1LI1, DYRK3, E2F3, E2F5, ECHDC3, EDEM3, EDIL3, EEA1, EEFSEC, EFNB1, EFNB3, EI24, ELL2, ELMOD1, ELMSAN1, EMC1, EME1, EML5, ENAM, EPHA4, EPS15L1, ERBB2, ERC1, ERGIC1, ESRRA, ESYT3, EVI5L, F2RL2, F8, FAM104A, FAM117B, FAM120AOS, FAM126B, FAM162B, FAM167A, FAM183BP, FAM207A, FAM208A, FAM212B, FAM46A, FAM71F1, FAM76A, FAM83A, FAT3, FAT4, FBXL19, FBXO10, FBXO16, FBXO41, FETUB, FGD6, FGF23, FGFRL1, FICD, FKBP1B, FKBP8, FLOT2, FN3KRP, FNDC3B, FNDC8, FOSL1, FOXJ2, FOXN2, FOXN3, FOXP1, FOXP2, FOXP3, FOXRED2, FPGS, FRA10AC1, FRMD4A, FRMD5, FUCA2, FUK, FUT11, FUT8, FUT9, FXYD2, FZR1, GAB1, GABRA3, GALNT7, GAS1, GATA3, GDAP1L1, GFRA1, GHDC, GIGYF1, GINS3, GK5, GLCE, GLYATL3, GMFB, GMIP, GMNC, GMNN, GNAI2, GNAO1, GNPDA1, GOLPH3L, GORASP2, GPALPP1, GPR12, GPR156, GPR158, GPR17, GPR22, GPR85, GPS2, GREM2, GRID1, GRK6, GRM7, GSDMB, GXYLT2, GYPE, HAAO, HCN3, HDAC1, HEXDC, HIRIP3, HLX, HMBOX1, HMGCS1, HNF4A, HNRNPA1, HNRNPUL2, HOOK3, HOXA13, HPCAL4, HPSE, HSPA13, HSPA1B, HSPB6, HSPBP1, HTR2C, HYAL3, IDH1, IDO2, IER5, IFI35, IFNLR1, IGFBP3, IGSF1, IGSF3, IKZF1, IL10RB, IL6R, INA, INHBB, INMT, INPP5K, IPO11, IPO11-LRRC70, IPO4, IQGAP3, IRAK2, IRGQ, ISLR, ITGA11, ITGB3, ITPR2, ITSN1, JADE2, JAG1, JAKMIP1, JARID2, JAZF1, JCAD, JMJD1C, JPH1, KBTBD6, KCND3, KCNH2, KCNH7, KCNJ8, KCNK9, KDM5D, KDM7A, KIAA1210, KIAA1211L, KIAA1217, KIAA1324, KIR2DL4, KIT, KLF4, KLF6, KLF7, KLHDC10, KLHDC3, KLHDC8B, KLK13, KLRD1, KLRK1, KMT2D, KRI1, KRT5, KRT74, LDAH, LDHA, LEF1, LELP1, LGI1, LGR4, LHPP, LHX2, LILRA1, LIMA1, LIMD2, LIN54, LMAN1, LMAN2L, LMBR1L, LNPK, LOC101928718, LOXL3, LPCAT1, LPO, LRIG1, LRRC40, LRRC46, LRRC55, LRRC7, LRRTM2, LYST, LZTS2, LZTS3, MAF1, MAGEA12, MAGEA2B, MAGEA3, MAP1A, MAP2K3*, MAP3K11, MAP4, MAP4K4, MAP7D3, MAPK1IP1L, MAPT, MARCH5, MARCH8, MARCKSL1, MARVELD2, MAST3, MAZ, MBD2, MBD6, MBLAC1, MBLAC2, MC2R, MCFD2, MCIDAS, MCOLN2, MCTP1, MDM4, MECR, MED8, MEDAG, MET*, METAP1, MEX3C, MFAP4, MFHAS1, MFSD8, MGAT4A, MGAT5B, MIGA1, MIGA2, MKLN1, MKNK2, MLLT1, MLLT3, MLST8, MMAB, MMP25, MOAP1, MORN4, MPP2, MPPED2, MRAS, MRNIP, MRPL33, MRPL52, MRTO4, MS4A2, MSL2, MTA2, MTMR4, MTMR9, MTNR1B, MTUS1, MYADM, MYCN, MYH9, MYNN, MYO1C, MYOCD, MYRIP, NAA50, NAP1L5, NAPEPLD, NAPG, NAV1, NAV3, NCBP2, NCDN, NCEH1, NDC1, NDRG1, NDST1, NDUFB5, NEAT1, NECTIN1, NECTIN2, NEDD8, NEUROD2, NFE2L1, NFKBIA, NFKBIZ, NISCH, NOL10, NOP2, NOS1AP, NOTCH1, NOTCH2, NOTCH3, NPTX1, NQO1, NR4A2, NRIP3, NRN1, NRXN2, NSD1, NTRK3, NUDT13, NUDT3, NUDT8, NUMBL, NUP210, NUP43, OAZ2, OLFML1, OLIG3, OR2H1, ORAI3, OSGIN2, OXSR1, P2RY14, PACS1, PAFAH1B2, PALLD, PALM3, PAPLN, PAX5, PCBP4, PDE4B, PDE7B, PDGFRA, PDXK, PDZRN3, PEA15, PEG10, PER2, PFKM, PGF, PGM1, PGRMC2, PHF19, PHF24, PHKG2, PID1, PIGZ, PIP4P2, PIP5K1A, PITPNC1, PKIA, PKP4, PLA2G15, PLA2G2D, PLA2G2F, PLAG1, PLCB1, PLCG1, PLCXD3, PLEKHH2, PLIN4, PLN, PLOD1, PLPPR2, PMF1, PNOC, PNPLA3, PNPLA8, PODXL, POGZ, POLL, POMZP3, POTED, POU2F1, POU4F1, POU6F1, PPARGC1B, PPFIA1, PPIL2, PPM1A, PPM1M, PPP1R10, PPP1R11, PPP1R14D, PPP1R16B, PPP1R8, PPP2R3A, PPP2R5A, PPP6C, PRAP1, PREB, PRELID2, PREPL, PRKAA2, PRKACB, PRKAG1, PRKCQ, PRKD1, PROX1, PROZ, PRRG3, PSD3, PSD4, PSMD5, PTBP3, PTGIS, PTPN2, PTPN4, PTPRD, PTPRJ, PTPRM, PTRH1, PURB, PXDC1, RAB11FIP2, RAB11FIP4, RAB3B, RAB3C, RAB43, RAD9A, RAET1L, RAI14, RALGDS, RALGPS2, RALY, RANBP10, RAP1GAP, RAP1GDS1, RARB, RARG, RASGRP4, RASL12, RBM23, RBP5, RCAN1, RDH11, RDH12, RDH13, RECK, RELL2, RELN, RET, RFX3, RGP1, RGS17, RGS4, RHOBTB3, RIMS3, RNASEL, RNF128, RNF165, RNF170, RNF34, RNF4, RNF41, ROCK1, ROGDI, RORA, RPL31, RPS6KA4, RPS6KL1, RRAGC, RRAGD, RRAS*, RSPO4, RTF1, RTL6, RTL8A, RTL8B, RTN4RL1, RWDD2A, RXFP1, RXYLT1, S1PR3, SAMD12, SAR1A, SARDH, SATB1, SATB2, SCLT1, SCML2, SCN1A, SCN2A, SCN2B, SCNN1D, SDK2, SDR9C7, SEC61A1, SEH1L, SELPLG, SEMA4B, SEMA4C, SEMA4F, SEMA5B, SEMA6A, SENP3, SEPT3, SERPINB2, SERPINE1, SERPINF2, SESN2, SF3B3, SFT2D1, SGPP1, SGSM2, SGTA, SH3BGRL2, SH3PXD2B, SHANK3, SHISA7, SHISAL1, SHKBP1, SHMT1, SHOC2*, SIDT1, SIDT2, SIPA1, SIRT1, SIRT6, SIX3, SKI, SLC10A2, SLC12A2, SLC15A5, SLC22A12, SLC25A27, SLC25A44, SLC27A4, SLC29A1, SLC2A13, SLC30A3, SLC35G2, SLC44A2, SLC4A2, SLC4A7, SLC6A1, SLC6A17, SLC7A10, SLCO3A1, SMAD4, SMCO1, SMIM15, SMIM30, SMPD1, SMU1, SNAI1, SNCG, SNED1, SNX12, SNX13, SNX15, SNX17, SNX30, SNX4, SNX9, SOCS4, SOX4, SOX6, SPAM1, SPATA20, SPATS2L, SPCS2, SPICE1, SPRR2G, SPSB4, SPTBN2, SRC, SRPRA, SSBP2, SSX5, STAB2, STAC2, STC1, STK35, STK38L, STPG4, STRAP, STRN3, SUCO, SULT4A1, SUPT6H, SURF4, SVOP, SWT1, SYNGR2, SYNJ1, SYT1, SYT11, SYT4, SZRD1, TAF4B, TAF5, TAGLN, TANC2, TANGO2, TARBP2, TBC1D2, TBC1D25, TBC1D30, TBCK, TBL1XR1, TBRG1, TCF12, TDRD6, TEDDM1, TENM1, TEX264, TFDP2, TGFBI, TGFBR2, TGIF2, THEM5, THSD4, THUMPD3, TK1, TLX2, TM9SF3, TMCC3, TMEM109, TMEM141, TMEM164, TMEM167A, TMEM173, TMEM184B, TMEM200B, TMEM213, TMEM246, TMEM25, TMEM255A, TMEM273, TMEM35A, TMEM79, TMSB10, TMTC3, TMUB2, TNRC18, TNRC18P2, TNRC6B, TNS2, TOB2, TOM1, TOX, TP53INP2, TP53TG3, TP53TG3B, TP53TG3C, TPCN2, TPD52, TPD52L3, TPPP, TRAFD1, TRANK1, TRAPPC6A, TRARG1, TRIM21, TRIM41, TRIM67, TRIQK, TRPV2, TSN, TSPAN11, TSPAN14, TSPAN18, TSR1, TSSC4, TTC19, TTLL7, TUFT1, TULP1, TUSC3, TWIST2, TXNIP, UBA1, UBE2G1, UBL4A, UBN2, UBP1, UBXN2B, UCN2, UCP3, UHRF2, ULBP2, UNC119, USF1, USP31, USP54, UST, VAMP2, VASN, VAT1, VAV3, VCL, VPS37B, VPS37D, VPS39, VTCN1, VTI1A, VWA5B2, WASF1, WDCP, WFDC5, WIPI2, WISP2, WNT9B, WSCD2, XBP1, XBP1P1, XIRP1, XPO5, XRCC1, XYLT1, YDJC, YKT6, YTHDC1, ZBTB46, ZBTB5, ZBTB9, ZC3H12B, ZC3H4, ZCCHC17, ZDHHC15, ZDHHC16, ZDHHC17, ZDHHC23, ZER1, ZFHX4, ZFP2, ZHX2, ZIC5, ZMYM4, ZMYND11, ZNF16, ZNF207, ZNF281, ZNF282, ZNF285, ZNF304, ZNF324, ZNF449, ZNF501, ZNF551, ZNF561-AS1, ZNF614, ZNF623, ZNF641, ZNF644, ZNF672, ZNF821, ZNHIT1, ZNRF3, ZRSR1, ZSCAN9, ZYG11B |
| hsa-miR-499a-3p | ABCA8, ABHD12B, ABL2, ABLIM1, ACOT2, ACTL6B, ACTR8, ADAMTS9, ADGRG6, AFF3, AGXT2, AK2, ALPK2, AMMECR1, ANKDD1B, ANKMY2, ANO5, AP3M1, AQP11, ARHGAP18, ARID1A, ARID1B, ARL8A, ARMCX2, ARMT1, ASB3, ASPH, ATP5PF, ATP6AP2, ATP6V1C1, ATP6V1E1, ATP8A1, ATXN7, B3GNT2, B4GALT6, BAZ2A, BCL11A, BCL2L14, BCLAF1, BDP1, BIRC8, BLCAP, BMF, BMP10, BMPR1A, BMT2, BRD1, BTLA, C4orf46, C5orf30, C5orf63, C7orf31, C9, C9orf40, CACHD1, CACNA1B, CACYBP, CADPS2, CAPRIN2, CAVIN2, CCDC137, CCDC7, CCDC96, CCL8, CCNL1, CCSER1, CD244, CD93, CDC42EP3, CDK5R1, CENPC, CEP44, CHD7, CHP1, CIAPIN1, CLDN22, CLEC7A, CLN8, COG4, COL4A3BP, COMMD8, COP1, COX7A2L, CPD, CPEB2, CPEB3, CPTP, CREB1, CREB5, CRISPLD1, CSMD3, CSTF1, CXCL8, CXXC4, CYB561D1, CYB5A, CYP26B1, DACH2, DAZ3, DAZAP2, DCAF12L2, DCAF8L1, DCUN1D4, DDA1, DDX17, DDX3X, DLGAP1, DLGAP2, DLL1, DMXL2, DNAH8, DNAH9, DR1, DRAM1, E2F6, E2F8, EDEM3, EED, EGLN3, EGR2, ELAVL2, ELK4, EMCN, EML4, EPC2, ESRRG, ETF1, ETFBKMT, ETNK1, EWSR1, EYA1, FABP4, FAM117B, FAM126B, FAM199X, FAM214A, FAM49B, FAM89A, FCHO2, FCRL4, FEM1B, FGFBP3, FHL1, FIGN, FMR1, FN1, FOXN2, FOXN3, FOXO1, FRY, FXYD6, GABRB3, GALNT3, GATAD2A, GFI1, GFRA1, GIMAP8, GLO1, GLYCTK, GMNC, GNG2, GNPTAB, GON7, GPR65, GPRASP1, GRAMD1C, GRM3, GSPT1, GSTCD, GUCY1A1, GXYLT1, H3F3B, H3F3C, HAUS5, HEATR5A, HELZ, HESX1, HHLA3, HK2, HMGB3, HNRNPDL, HNRNPF, HOXA5, HOXC4, HSPA1L, HUS1, IGFBP4, IL19, IL1RN, IL20, INO80D, IPO8, ISG20, IVNS1ABP, JADE2, JAK2, JMJD1C, KAT2B, KBTBD2, KCNE4, KCNJ14, KCNJ3, KIAA0040, KIF1B, KIF1BP, KLHL15, KMT2C, KSR1, LAMB3, LAMP2, LEPROT, LETMD1, LIAS, LIFR, LIMA1, LIN54, LMBRD2, LRFN2, LRP2, LYRM1, MAF, MAML3, MAMLD1, MAN1A1, MAP3K1, MAP3K2, MAP4K3, MAPK1, MAPK8, MAPK8IP3, MAPK9, MARCKSL1, MBNL2, MCMDC2, MDM4, MEF2C, MEOX2, MFN2, MIER1, MIER3, MOCS2, MPP7, MRAS, MRPS12, MSANTD2, MSL2, MSRB3, MTPN, MUT, MYO1D, MYO6, MYRFL, NAA15, NAA50, NAPG, NAT8L, NCKAP1, NCOR1, NDFIP1, NECTIN2, NEK2, NFE2L2, NFIL3, NHLH2, NKX3-1, NNT, NPAT, NRIP1, NRSN1, NSA2, NT5C3A, NTRK2, NUFIP2, NUS1, NXN, NXPE2, OAZ2, ODF4, OMG, OTUD1, OTUD4, OTUD6B, OXCT1, PAK3, PAPD5, PAPOLB, PAPPA-AS1, PATZ1, PCDH19, PCDH7, PCDH9, PCLO, PCM1, PCSK2, PDE12, PDE6A, PDGFC, PDHA1, PDPK1, PDS5A, PELI1, PGAM1, PGAM4, PGAP1, PHC3, PHLPP2, PI15, PIAS2, PITPNA, PLCG1, PLEKHB2, PLEKHG5, PLPPR1, PLS3, PNISR, POM121, POM121C, PPM1B, PPM1D, PPM1E, PPP1R26, PPP2R1B, PPP4R4, PRKAG2, PRKD3, PRKG1, PRKX, PROSER2, PRPF40A, PRR13, PSMA8, PSMG4, PTGFRN, PTMA, PTP4A1, PTPN4, PTPRA, PUM2, PURB, QRFPR, RAB2B, RAN, RANBP9, RAP2C, RAPGEF2, RARB, RARS2, RASA1*, RASSF2, RASSF8, RB1, RBM27, RCHY1, RCL1, RCOR1, REEP3, RELN, RETREG1, RFLNB, RFX2, RNF144A, RNF2, RNF4, RNMT, RSAD2, RSBN1, RTL5, RUFY3, RUNX1T1, RUNX2, RWDD1, RWDD4, SAE1, SCRIB, SDCBP, SEC62, SEMA6A, SEPT7, SFRP4, SGPP1, SH2D1B, SHISA7, SHPRH, SLC17A5, SLC17A6, SLC25A40, SLC2A13, SLC36A3, SLC7A2, SLC9A6, SLFN12, SLITRK1, SLMAP, SMIM13, SMIM21, SMOC2, SNAP25, SNX3, SOGA3, SON, SOX11, SOX6, SP4, SPATA2, SPIN1, SPOCK1, SPRR2G, SPTBN1, SPTSSA, SRRM1, SRSF7, SRXN1, STIM2, STK17B, STPG1, STX12, STX1B, STXBP5L, STYX, SUCO, SUGP2, SYNCRIP, SYNPR, SYT6, TAF9B, TANC1, TAOK3, TBC1D22A, TBX3, TCAF1, TCF7L2, TEF, TENM3, TENM4, TET2, TEX30, TFCP2, THRB, TM9SF3, TMEFF1, TMEM178B, TMEM255A, TMEM260, TMEM38B, TMEM9B, TMSB10, TMX1, TOLLIP, TOR1A, TOX, TRAF5, TRAPPC11, TRDN, TRIB2, TRIM59, TRMT9B, TRPS1, TSC22D3, TSGA10, TSNAX, TTC39A, TTF2, TTN, TWSG1, TXNIP, UBE2D3, UBE2E3, UBE2G1, UBFD1, UBN1, UBR3, UEVLD, UHMK1, UNC5D, UQCRB, USP13, USP6, USP9X, VANGL1, VIP, VPS35, VRK1, VTI1A, WARS, WASF3, WDR76, XIAP, ZC2HC1B, ZC3H6, ZDHHC17, ZDHHC21, ZEB2, ZFAND5, ZIC2, ZIC3, ZIC4, ZMAT4, ZMYND8, ZNF16, ZNF181, ZNF225, ZNF24, ZNF257, ZNF260, ZNF423, ZNF460, ZNF529, ZNF580, ZNF667, ZNF99, ZNRF1, ZSCAN16, ZZZ3 |
| hsa-miR-499b-5p | ABCC5, ACIN1, ACSS1, AGRN, AGTPBP1, AHI1, AHSA2, AMD1, AMMECR1L, ANKRD55, APBB1, APOB, APOBEC3H, ARHGEF10L, ARL4C, ATF2, B2M, B4GALT6, B4GAT1, BASP1, BCO1, BCORL1, BDNF, BIN2, BPIFC, BSPRY, C17orf75, C18orf25, C2CD2, C3orf80, C5orf22, C5orf24, C7orf33, CADM4, CALD1, CAMSAP2, CBFB, CCDC107, CCDC82, CCNI, CD2AP, CDK6, CEACAM5, CHD6, CNGA4, CNOT8, CNOT9, COL17A1, COL25A1, COL9A2, COLEC10, COPS3, CRIPT, CRISP3, CTPS2, CUL1, DDX1, DDX4, DERL2, DHX32, DKC1, DLG2, DMD, DNAJB11, DNER, DPY19L3, DRGX, EIF1AX, EIF5, ELF2, EN2, ENO2, EP300, EPC1, F2R, FADS3, FAM111A, FAM117B, FAM136A, FAM193A, FAM199X, FAM19A1, FAM53C, FBXO40, FER, FLRT3, FNDC3A, FOXD3, FRMD5, FRS2, FRYL, FSTL5, GABRG1, GALP, GCC2, GDF15, GDF6, GDNF, GGA2, GINS2, GLRB, GLYATL2, GNA12, GNB5, GORAB, GPR162, GRIN3A, GYG1, H3F3B, H3F3C, HAND1, HECW2, HEG1, HIST1H2BD, HIVEP3, HLA-DQA1, HRNR, HSP90AA1, HSPH1, IER3, IFNGR1, IFRD1, IFT80, IGSF5, IKBIP, IL20RB, INAVA, ISL1, ITCH, ITIH5, JAM3, JAZF1, KATNAL1, KCNJ3, KCNMB2, KCTD2, KCTD6, KIAA0586, KIAA1109, KLRC4, KMT2B, KMT5B, KNTC1, KRTAP2-1, KYNU, LACTB2-AS1, LARP4B, LITAF, LMLN, LONRF2, LONRF3, LRBA, LRRC71, LTB4R, LY6K, LYSMD3, MACO1, MAGEB18, MAIP1, MAPK6, MARK3, MBD4, MBNL2, MCHR2, MECR, MED23, MGAT3, MICU1, MINDY2, MINOS1, MKNK2, MMP2, MPLKIP, MRPL37, MRPS35, MRS2, MTMR2, MTMR3, MTPAP, MYBL1, NAALAD2, NAPG, NBPF8, NCBP2, NDFIP1, NEK2, NFAM1, NIPA2, NKTR, NKX3-1, NPLOC4, NTRK2, NUDT10, OCLN, OLIG1, OTOR, OTUD1, PARD6B, PCDHA1, PCDHA11, PCDHA2, PCDHA3, PCDHA4, PCDHA5, PCDHA6, PCDHA7, PDS5B, PEX10, PHACTR2, PHB, PHLDA1, PIGG, PLPP6, PLSCR1, PNN, PNO1, POP4, PPARGC1B, PPAT, PPP1R9A, PPP3R1, PRKCI, PRKD3, PRMT6, PRPF40A, PRR30, PTPRT, RAB11FIP2, RAD1, RASSF6, RHOV, RNF145, RNF149, RNF214, RPL37A, RPS6KL1, RRN3, RSBN1, RSRC1, RWDD4, SALL1, SCARB2, SCD, SCGN, SDC2, SEL1L, SEMA4B, SENP6, SENP7, SEPHS1, SERF1A, SERF1B, SERPINA3, SERTAD3, SESN1, SH3YL1, SHISA6, SLC12A2, SLC15A5, SLC16A10, SLC18A2, SLC22A3, SLC25A48, SLC2A10, SLC35D3, SLC35E2B, SLC35G3, SLC39A14, SLX1A, SMC6, SMIM9, SNX31, SOX4, SOX6, SRSF6, SUCO, SVOPL, TAF1, TAOK1, TARSL2, TCF4, TDG, TET1, TFCP2L1, TFIP11, TGFB2, TLDC1, TMEM169, TMEM189-UBE2V1, TMEM2, TMEM267, TMEM78, TMF1, TMX1, TOE1, TOPBP1, TP53INP2, TP53TG3, TP53TG3C, TRIM33, TSC22D2, TSHZ1, TSPAN12, TSSK4, TWIST1, TWSG1, TXK, UBA5, UBE2D3, UBE2E1, USP15, USP3, VPS13B, VPS45, WDR47, WDR77, ZBTB26, ZDHHC6, ZDHHC9, ZFP91, ZFYVE16, ZKSCAN2, ZMAT5, ZNF207, ZNF24, ZNF385A, ZNF449, ZNF529, ZNF582, ZNF629, ZNF703, ZNF845 |
| hsa-miR-548l | AADAC, ABCG2, ABHD10, ABHD13, ABHD5, ABI3BP, ACER3, ACPP, ACSL6, ACSM2B, ACTB, ACTN4, ACTR3, ACTR3B, ACVR2A, ADCYAP1, ADORA2B, AGO2, AGO4, AHCTF1, AHDC1, AKAP11, ALDH18A1, ALG9, AMFR, ANGPTL2, ANGPTL3, ANGPTL5, ANKRD12, ANKRD49, ANP32E, ANXA5, AP1AR, AP3B1, API5, APLP2, APOL4, APPBP2, APPL1, AQP11, ARID4A, ARL10, ARL13B, ARL2BP, ASCL1, ATAD2, ATF7IP, ATG16L1, ATG2B, ATMIN, ATP10B, ATP5G3, ATP6AP2, ATP6V0E1, ATP8A1, ATXN3, ATXN7, AUTS2, B2M, B3GAT2, BACH1, BACH2, BBS10, BBS12, BLOC1S6, BMI1, BMPR1A, BOLL, BRIX1, BRWD3, BTBD7, C11orf87, C3orf58, C4orf46, CA1, CAAP1, CANX, CAPZA1, CARF, CBLN4, CBR4, CBWD2, CBWD3, CBWD6, CCDC126, CCDC179, CCDC39, CCDC59, CCP110, CCSER1, CCT6B, CD164, CD28, CD46, CD47, CDC42SE2, CDCP1, CDK19, CDKL2, CDS1, CEP120, CEP41, CEP97, CFAP97, CFL2, CHD9, CHST6, CKAP2L, CLCC1, CLDN20, CLINT1, CNEP1R1, CNKSR2, CNOT6L, CNTN1, COA5, COG5, COL4A4, COL6A3, COX20, CPNE8, CPS1, CREBRF, CREBZF, CRK, CRYBG3, CSE1L, CSF2RB, CSNK1G3, CSNK2A1, CTCFL, CTHRC1, CTNNB1, CUL2, CUL4A, CXADR, CYP20A1, CYR61, DACT1, DCUN1D4, DDTL, DDX3X, DEK, DENND1B, DESI2, DHRS7, DHX29, DIAPH2, DLG2, DMXL1, DNAJB14, DRAM2, DYNLT1, E2F4, EDDM3B, EFCAB14, EFNA5, EGR2, EIF1AY, ELAVL2, ELOA, EMB, EML4, ENPP5, EP400, EPHA4, ERH, ERO1A, ESYT1, ETF1, ETNK1, ETS1, ETV3, EXO5, EXOC5, EXPH5, F3, FAIM, FAM133B, FAM135A, FAM169A, FAM177A1, FAM183A, FAM198B, FAM19A1, FAM19A4, FAM24B, FAM3C, FAM47E, FAM49B, FAM84B, FAM89A, FAM8A1, FAM91A1, FBXL3, FBXL5, FBXO28, FEM1C, FGD4, FGD6, FGF5, FGFBP1, FGL2, FILIP1L, FLJ21408, FLYWCH2, FOPNL, FOXF2, FOXK1, FOXN2, FOXP2, FUBP1, FZD6, GAS7, GATA2, GCNT2, GDE1, GEMIN6, GFM1, GIMAP1, GIMAP4, GMFB, GNG12, GOLGA8A, GOPC, GPATCH11, GPBP1L1, GPM6A, GPR155, GPR26, GRIK2, GRIP1, GSK3B, GTF2H5, H3F3A, HELQ, HHLA2, HIVEP2, HLTF, HMGB2, HMGCR, HNRNPH3, HOOK1, HOXD8, HPRT1, HSCB, HSP90AA1, ICA1L, IDI1, IGF2BP3, IGFBP5, IGIP, IGSF11, IL5, IL6ST, IMPAD1, INPP5A, IPMK, ITGB8, IYD, JADE3, JDP2, KATNAL1, KCNA1, KCNG3, KCNIP4, KCTD12, KCTD5, KHDC4, KIAA1586, KIF18A, KIF1B, KIF2C, KIF3A, KLRC3, KRAS*, LACC1, LACTB2, LEPROT, LGR4, LHFPL5, LIMS1, LINC01553, LINC01590, LIPA, LIPF, LNPEP, LRRC59, LRRC70, LRRC8B, LTV1, LYRM2, LYZL2, MAL, MANEA, MAP2K1*, MAP3K20, MAPK8, MARCH1, MARCH7, MARCKS, MBD2, MBIP, MBLAC2, MDFIC, MDM4, MED13, MEGF11, MFSD9, MIEF2, MIS12, MKLN1, MOB1B, MOB4, MOCS2, MRPS36, MRPS5, MSANTD2, MTCP1, MTF1, MTHFD2, MTMR10, MTPN, MTR, MUT, MYCN, MYF5, MYLIP, MYO5A, MYO5B, MYSM1, N4BP2, NAA25, NACC2, NADK, NBEA, NCAM1, NCAPD2, NDC1, NDST4, NECTIN2, NEGR1, NEK1, NEK7, NENF, NEXMIF, NFATC3, NFE2L2, NFIA, NFKB1, NKTR, NLGN1, NLN, NOVA1, NQO1, NR1I2, NSRP1, NT5E, NUCB2, NUCKS1, NUDT11, NUP133, NUP160, NUP37, ODAPH, OSTC, OTUD1, PACSIN2, PAFAH1B2, PAIP1, PAK5, PAPPA, PARP11, PARP15, PAWR, PCDHA6, PCGF5, PDCD6IP, PDE12, PDE4D, PDIK1L, PELI1, PGM2, PHACTR2, PHACTR3, PI4K2B, PIBF1, PIK3C2A, PKN2, PLEKHG1, PLEKHG7, PLPP5, PNO1, POLR3G, PPARGC1B, PPEF2, PPHLN1, PPIC, PPIL4, PPM1A, PPP1R12A, PPP2R5E, PPP6C, PRELID1, PRKAA2, PRKAR2B, PRKD3, PRKG1, PRKG2, PRLR, PSAT1, PSMA1, PSMA4, PTGES3L, PTP4A1, PTPN13, PUM1, RAB32, RAB39B, RAG1, RAI14, RAP1A, RAP1B, RAP2C, RAPGEF2, RASA1*, RASA2*, RBFOX2, RCC2, REEP3, REST, REV3L, RFC3, RFC5, RHD, RICTOR, RNF111, RNF138, RNF146, ROBO1, ROBO2, RORA, RP2, RPE65, RPGRIP1L, RPRD1A, RPS15A, RPS6KA3, RRAS2*, RRN3, RWDD4, RXYLT1, RYBP, S100A7A, S1PR1, SACS, SAMD8, SAR1A, SAR1B, SAXO2, SCAF11, SCN8A, SDAD1, SEC61A2, SELENOT, SEM1, SEPT2, SERBP1, SESTD1, SETD7, SGIP1, SH2D1A, SH3BGRL, SH3RF1, SHISA9, SIMC1, SIPA1L2, SKI, SLAIN1, SLC15A5, SLC25A21, SLC25A24, SLC25A36, SLC25A44, SLC36A4, SLC38A2, SLC45A4, SLC4A7, SLC5A3, SLC6A15, SLC9A2, SLC9A4, SLK, SMAD4, SMAD5, SMG1, SMIM15, SMIM8, SMKR1, SNHG28, SOCS6, SORCS2, SP1, SP4, SP5, SPACA1, SPAG6, SPATA13, SPC25, SPCS3, SPO11, SPOCK1, SPOPL, SPPL2A, SPRED1*, SPRYD7, SQOR, SRSF10, SRSF6, SS18, SS18L1, SSU72, ST8SIA1, STAG2, STK17B, STK39, STXBP5, SUB1, SUPT7L, SYCP2, TAF2, TBCA, TCF7L2, TEX12, TFAM, TGOLN2, TM9SF3, TMEM170B, TMEM241, TMEM254, TMEM30A, TMEM30B, TMEM41B, TMEM56, TMOD2, TMPRSS11D, TMPRSS7, TNFAIP6, TNIP2, TOM1L1, TOR1AIP2, TOX3, TPBG, TPD52L3, TPRA1, TRIM36, TRIP11, TRIQK, TRMT13, TRNAU1AP, TRUB1, UBASH3B, UBE2H, UBE2K, UBE3D, UFL1, UHMK1, UNC80, URI1, USP12, USP25, USP33, USP37, USP44, USP6NL, UST, UTP3, VAV3, VGLL3, VIM, VMP1, VPS29, VPS54, WAPL, WDR11, WDR26, WTAP, XIAP, XKR9, XPO4, XPOT, XRCC6, YAP1, YOD1, YPEL2, YWHAE, ZBTB20, ZBTB33, ZBTB37, ZBTB43, ZBTB5, ZBTB6, ZCCHC14, ZDHHC20, ZDHHC21, ZEB2, ZFAND1, ZFPM2, ZIK1, ZKSCAN7, ZMIZ1, ZNF12, ZNF148, ZNF257, ZNF282, ZNF354C, ZNF460, ZNF470, ZNF493, ZNF562, ZNF624, ZNF678, ZNF711, ZNF724, ZNF770, ZNF800, ZNRF2 |
| hsa-miR-575 | A4GNT, ABCD3, ABHD14B, ABHD17B, ABHD5, ACOX1, ACP2, ACTL6A, ACTR3B, ADAMTSL5, AGAP2-AS1, AIMP1, AKAP13, AKNA, ALG14, ANKRD23, ANKRD46, ANKRD54, API5, APOBEC4, ARHGAP21, ARHGEF38, ARID1B, ARV1, ASCC1, ATAD5, ATL2, ATP2A2, ATP6V1G2, BBX, BCL2L1, BCL9, BID, BLID, BLOC1S3, BNIP3L, BRMS1L, BRWD3, BTG2, C10orf71, C15orf52, C20orf144, C21orf2, C3orf58, C8orf76, CA13, CACNA2D1, CADM2, CAMK2N2, CAPS, CCDC138, CCDC66, CCDC69, CCNA2, CCND3, CCS, CD300LG, CD40, CD68, CDC27, CDC42EP3, CDC45, CDC7, CDH24, CEP128, CETN1, CHCHD4, CHD9, CHRD, CHST6, CHSY3, CLPP, CMTM4, CNEP1R1, CNKSR3, CNNM4, CNPY2, CNTN1, COL24A1, COMT, COX15, CPNE2, CRCP, CREB5, CSMD3, CSNK1G3, CSRNP1, CSTA, CSTL1, CXCR1, DAZAP1, DBN1, DCAF16, DCTN3, DCUN1D4, DDT, DENND1C, DENND5A, DENND6A, DGKH, DHX33, DLC1, DMGDH, DNAH3, DNAJB14, DNAJC13, DNM1L, DPP8, DTD2, DUSP2, DYDC1, DYNAP, DYNC1LI2, EDIL3, EGFR, ELAVL4, EPB41L5, EPC2, EPCAM, EPHB1, EPOR, EXPH5, FAM118A, FAM19A1, FAM222B, FAM234B, FAM81A, FAM89A, FAM92A, FAM98A, FAS, FAT3, FBXO15, FBXW11, FGF1, FIP1L1, FJX1, FKBP14, FLNB, FMO1, FOXN4, FOXRED1, GALNT1, GCLC, GCM2, GDF5OS, GDI2, GRAMD2A, GRIA2, GRK2, GRK4, GRWD1, GUCY2C, GYPE, HDAC7, HIP1, HORMAD1, HPD, HSD11B2, IARS2, ICA1L, IFNLR1, IGLON5, IL15, ITPRIPL2, JADE2, KCNA1, KCNIP2, KIAA1324L, KIF1C, KIF2C, KIR2DL1, KIR2DL3, KIR2DL4, KIR3DL1, KIR3DL2, KIR3DL3, KIR3DX1, KLF6, KLHL29, KLHL6, KREMEN1, KRT75, LEMD3, LIX1L, LMBRD1, LPXN, LRFN5, LRP1, LRRC10B, LRRN3, LRTOMT, LSG1, LSP1, MANSC1, MAP3K1, MAPKAPK3, MAVS, MB, MDM4, MEAF6, MED10, MED28, MED29, MEF2C, MEF2D, MEIG1, MFAP4, MFSD13A, MFSD14A, MKLN1, MMP15, MRPL49, MRPS16, MRPS23, MRPS36, MS4A4A, MSRB3, MXD1, MYH10, MYL1, MYO10, MYOZ2, N6AMT1, NANOGNB, NAP1L6, NCAPG, NCBP3, NDEL1, NEMP1, NENF, NEURL1, NEUROG1, NFAT5, NOLC1, NSMCE2, NUTM1, NUTM2G, OFD1, OR51E2, ORAI2, OTUD5, OTULIN, P2RY14, PARP9, PCDH19, PCLO, PEX5L, PGAP1, PGPEP1, PIGS, PITX3, PKD1L2, PLCG2, POLD3, POLR2F, POLR3B, POU5F1, PPIE, PPP1R14D, PPP1R3B, PRKX, PRSS46P, PTCD2, PTEN, PTGES2, PTPRF, PUM2, PURB, PYM1, QPCTL, RAB25, RAB38, RAB3GAP2, RACK1, RAD51, RB1CC1, RBBP5, RBFOX2, RBM23, RBP2, RDH10, REG4, REPIN1, RFX5, RGS3, RHOV, RIOK3, RIPK4, RLF, RNF166, RPUSD1, RSBN1, SACS, SBNO1, SCAF8, SEH1L, SEPT4, SERBP1, SGCD, SHISA3, SHOC2*, SHROOM4, SIK2, SLAMF9, SLC24A3, SLC25A32, SLC35F1, SLC43A1, SLC4A1AP, SLC6A6, SLC7A7, SMG1, SMIM12, SMU1, SNAPIN, SNRNP40, SNRNP48, SNRPD3, SNX11, SOWAHC, SPAST, SPEN, SPRYD3, SRR, SRRD, SRSF2, ST7L, ST8SIA1, STAT5A, STIL, STK4, STOX1, SURF4, SYNPO2L, SZRD1, TESPA1, TFCP2L1, TFPI, TIMM29, TLE4, TLN2, TMCC1, TMEM177, TMEM237, TMEM263, TMEM81, TMEM9B, TMSB4Y, TOP2B, TOX, TPT1, TRAF3IP1, TRAF3IP2, TRAF4, TRIM22, TRIM59, TRIM7, TSG101, TSPAN17, TSPO2, TSSK1B, TTC22, TUFT1, UBAP2, UBN2, UBXN2A, UEVLD, UFD1, UMPS, USE1, USP1, UTP14A, VASH2, VASP, VPS36, VPS39, VTI1A, WDFY3, WDR76, WDR92, WEE1, WFDC9, WIPI2, WNK1, WSB2, XIAP, XPNPEP3, YPEL5, ZBTB33, ZC3H12A, ZCCHC4, ZDHHC21, ZFAND2A, ZFP1, ZFP64, ZNF212, ZNF318, ZNF329, ZNF425, ZNF471, ZNF48, ZNF516, ZNF556, ZNF584, ZNF609, ZNF677, ZNF680, ZRANB3 |
| hsa-miR-593-5p | ABCE1, ABCF1, ABHD15, ACOT11, ADAM19, ADAMTS16, ADCY5, ADCY6, ADGRF2, ADSS, AEBP1, AGAP3, AHCY, ALPK3, ALS2, AMD1, ANKRD36, ANKRD40, ANKRD44, ANO10, AP4S1, APCDD1L, APLNR, ARFGAP2, ARHGAP19, ARHGAP21, ARHGEF12, ARID3B, ARL4D, ARL6IP1, ARL8B, ARMC7, AS3MT, ASAP2, ATP2A3, AVL9, AWAT2, AZIN1, BBS12, BBS5, BCAR1, BCAT1, BCL11B, BCL2L2, BIRC5, BRWD1, BTRC, C11orf72, C16orf87, C19orf12, C1orf220, C2CD4A, C6orf106, C8orf31, CA6, CANT1, CAPN12, CARHSP1, CARNMT1, CAV1, CBY1, CCDC126, CCDC144NL, CCND1, CCND2, CCS, CD164, CD99L2, CDC25B, CDC42SE2, CDCA7L, CELF1, CELSR3, CEP170B, CHIC2, CKB, CLEC2D, CLK3, CLNS1A, CLU, CNEP1R1, CNGB3, CNP, COL11A1, COL13A1, COLEC11, COMMD7, CORO2A, CSMD1, CSNK1D, CSTF3, CTDSP1, CTDSPL2, CYBRD1, DACT1, DAZAP2, DCLRE1C, DCUN1D1, DENND5A, DLX5, DNLZ, DNTTIP2, DOK1, DOPEY1, DPP8, DSC1, DSG2, DUS2, DUSP13, DUSP18, DUSP3, DZIP1, EDAR, EDNRA, EFNA4, EGR1, EGR2, EIF4A3, EIF4EBP2, EIF5, ELF3, ELMO2, ENPP4, EPB41L4B, EPS8L2, ERBB2, ETV1, EVPL, FAF2, FAM133A, FAM13B, FAM46C, FAM53C, FAM57A, FBXL12, FBXL5, FBXO32, FBXO41, FCF1, FGF9, FGFR1OP, FLG2, FLOT2, FMNL2, FN1, FOSL1, FOXK2, FOXO1, FOXO3, FOXP2, FRMD5, FRS2, FSTL3, FUT8, FZD5, G3BP2, GAB2, GATAD2B, GIT2, GJA4, GLOD4, GNB3, GNPNAT1, GOLGA6A, GOLPH3, GOSR1, GRAP2, GRASP, GRK2, GRM1, GRM5, GRSF1, GSPT1, GTDC1, HADH, HBEGF, HCN1, HDAC7, HEATR1, HMBOX1, HNMT, HNRNPA2B1, HNRNPDL, HP1BP3, HRH1, HS3ST5, HSPA4, HTR2C, IDH2, IKZF2, IL12RB1, IL16, IL17RD, IL24, INAVA, INPP5A, IST1, ITGB2, JPT2, KDELR3, KIAA1143, KIAA1217, KIDINS220, KIF13A, KIF18B, KIF3B, KLF6, KLHL24, KLHL25, KLHL42, KPNA3, KRT74, KRT80, LAS1L, LCK, LCLAT1, LDLRAP1, LMOD3, LNX2, LOC100128398, LPAR6, LRCH2, LRP11, LRRC31, LRRK1, LUC7L2, LY6E, MAOA, MAP3K12, MAP3K20, MAP3K8, MARCKSL1, METTL2B, MFF, MFSD4B, MICA, MIEF1, MIEF2, MIF4GD, MIPOL1, MMD, MMD2, MPP5, MPRIP, MSN, MYO18A, MYO1B, NAALADL1, NAXE, NCDN, NDEL1, NDST1, NEMP1, NEUROD4, NFAM1, NFYB, NHLRC3, NIP7, NKPD1, NKRF, NOD1, NOL9, NPAS3, NPTX2, NR3C1, NRG1, NSMCE3, NSMF, NTM, NUAK2, NUP153, NUP160, NXF1, NXN, OCIAD2, OCRL, OGFOD2, OGN, OGT, OPCML, ORAI2, OSBPL11, OTUD4, OVOL1, OVOL2, PAFAH1B1, PAM, PARP8, PATE4, PDCD7, PDE3A, PDE6C, PDZRN3, PELI2, PHF10, PHKA1, PI15, PIK3C2A, PIK3R3, PITPNM3, PIWIL1, PKIA, PKP1, PLEC, PLEKHF2, PLK1, PLK3, PLXNA4, PMP22, PPM1A, PPM1D, PPM1F, PPP1R14B, PPP1R15A, PRICKLE1, PRMT2, PRRC2C, PRUNE2, PRX, PSEN2, PSKH1, PSMB5, PTPDC1, PTPN4, PTPRO, PWWP2A, QPCTL, QPRT, QSER1, RAB11A, RAB43, RAD1, RAD51, RAD51D, RARG, RCOR1, RFC2, RFX3, RGMA, RGPD1, RHOG, RHOU, RHOXF1, RIMKLA, RIMKLB, RIMS4, RIPK4, RNF114, RNF165, RNF19A, RNFT1, ROBO2, RPL7L1, RPP14, RRM2, RRP12, RUFY2, S100PBP, SBK1, SCTR, SEC14L6, SERF1A, SERINC2, SERTAD4, SFT2D3, SGMS2, SGO1, SH2B3, SIKE1, SIPA1L2, SIRPA, SLC13A2, SLC24A4, SLC35B2, SLC47A1, SLC4A5, SLC7A8, SMAD5, SMAP1, SMIM15, SMOC1, SNHG28, SOCS5, SP1, SPINDOC, SPOCK3, SPOUT1, SPSB1, SRGAP3, SRRD, SRRM3, SRSF10, SSC4D, SSNA1, ST6GALNAC4, ST8SIA1, STK17B, STK26, STK36, STUM, TAOK2, TBC1D4, TCEANC2, TEDDM1, TERF2, TET1, TET3, TEX261, TFDP2, THSD4, THUMPD1, TIMP3, TLN1, TMED4, TMEM117, TMEM119, TMEM121B, TMEM129, TMEM214, TMEM267, TMPPE, TMUB2, TNIK, TPI1, TPP1, TRAF4, TRAM2, TRIM31, TRIM71, TRIM72, TRPM3, TRPS1, TSC22D3, TSEN15, TSHZ2, TSPAN14, TSPO, TSPO2, TSTD2, TTC26, TTC7A, TTC9C, TTYH3, TUBB, TUBB6, TUSC2, TWSG1, UBALD1, UBE2D4, UBE4A, UBIAD1, UBTD2, UNKL, UPB1, UPK2, VAC14, VAPA, VIPAS39, VKORC1L1, VSIG10L, VWA5B2, WDCP, WIPF1, WIPF2, XIAP, YDJC, YOD1, ZBTB44, ZC2HC1A, ZCCHC10, ZDHHC17, ZFPM2, ZKSCAN2, ZMYND19, ZNF395, ZNF420, ZNF512B, ZNF551, ZNF609, ZNF92 |
